# Plasma-Activated 3D-Printed Electrode for the Determination of Tolfenamic Acid in Environmental Samples

**DOI:** 10.1021/acsomega.6c08647

**Published:** 2026-10-29

**Authors:** Cassiano Cunha de Souza, Francisco Walison Lima Silva, Anne Alves Macedo, Dayenny Louise D̀Amato, Célia Machado Ronconi, Wallace Burger Veríssimo de Oliveira, Raquel G. Rocha, Lucas V. de Faria, Fernando Henrique Cincotto, Rodrigo Alejandro Abarza Muñoz, Maria Auxiliadora Costa Matos, Renato Camargo Matos

**Affiliations:** † Department of Chemistry, 28113 Federal University of Juiz de Fora, Juiz de Fora, Minas Gerais 36036-900, Brazil; ‡ Institute of Chemistry, 28125 Federal University of Rio de Janeiro, Rio de Janeiro, Rio de Janeiro 21941-909, Brazil; § National Institute of Science and Technology of Bioanalytics − Lauro Kubota (INCTBio-LK), Institute of Chemistry, University of Campinas, Campinas, São Paulo 13083-970, Brazil; ∥ Institute of Chemistry, Fluminense Federal University, Niterói, Rio de Janeiro 24020-141, Brazil; ⊥ Department of Fundamental Chemistry, Institute of Chemistry, University of São Paulo, São Paulo, São Paulo 05508-000, Brazil; # Institute of Chemistry, Federal University of Uberlândia, Uberlândia, Minas Gerais 38408-100, Brazil

## Abstract

The occurrence of tolfenamic acid (TA) in aquatic environments has raised concerns regarding its potential environmental impacts, highlighting the need for rapid, sensitive, and low-cost analytical methods for its determination. In this work, a conductive CB/PLA filament was fabricated through a solvent-free thermal mixing approach and used to produce a 3D-printed electrochemical sensor for TA determination. This strategy provides a greener and scalable route for electrode fabrication. Atmospheric air plasma treatment was applied to improve the electrochemical performance of the CB/PLA electrode. SEM images revealed the partial removal of the insulating PLA matrix, increasing the exposure of the conductive carbon sites. This modification resulted in lower charge-transfer resistance (*R*_ct_ from 33.4 to 7.6 kΩ). Under optimized differential pulse voltammetry conditions, the plasma-treated electrode (CB/PLA-PT) exhibited a linear response toward TA in the concentration range of 1.91–20.1 μmol L^–1^, with detection and quantification limits of 0.54 and 1.80 μmol L^–1^, respectively. The sensor demonstrated satisfactory precision (RSD ≤ 5.7%) and selectivity in the presence of common interfering species. Its applicability was successfully demonstrated in spiked river and tap water samples, yielding recovery values between 92 and 105%. The proposed approach combines 3D printing and reagent-free plasma activation to provide a simple and environmentally friendly platform for the electrochemical determination of TA in environmental waters.

## Graphical abstract

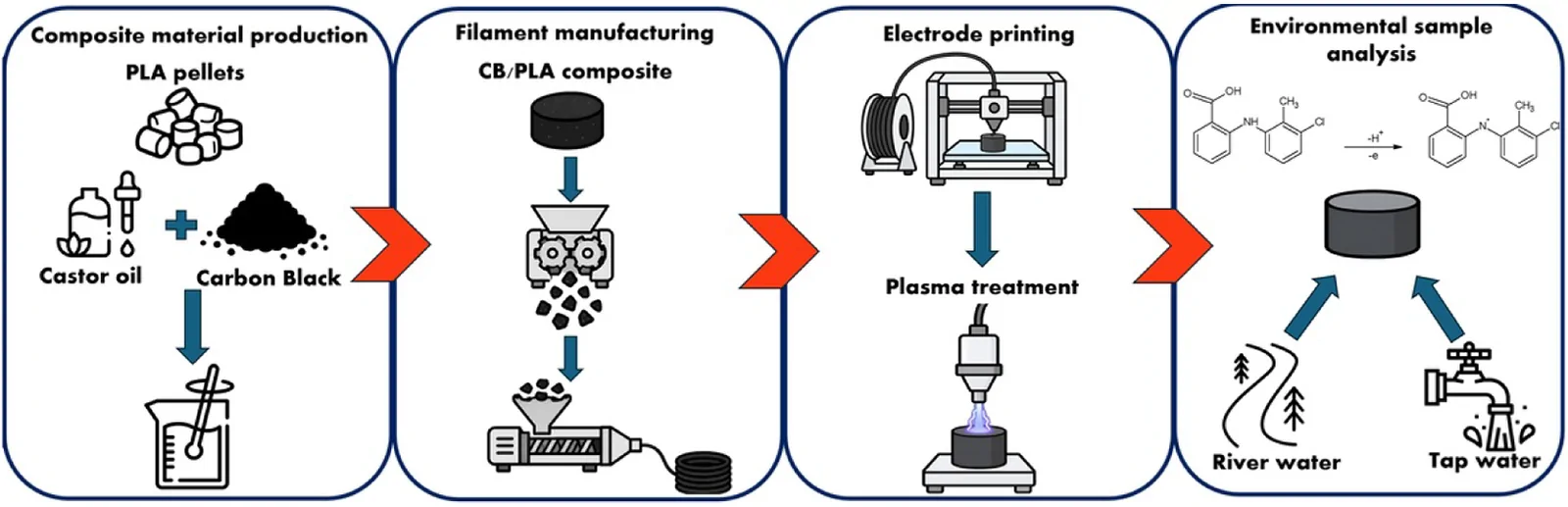

## 1 Introduction

Nonsteroidal anti-inflammatory drugs (NSAIDs) are among the most widely consumed pharmaceuticals worldwide.^1,2^ Their extensive use in human and veterinary medicine, combined with the incomplete removal by conventional wastewater treatment processes, has led to their frequent detection in environmental compartments such as surface waters, wastewater, sediments, and even drinking water.^2,3^

Among these pharmaceuticals, tolfenamic acid (TA) is a fenamate derivative commonly used for the treatment of migraine and inflammatory disorders in both human and veterinary medicine. Because of its widespread use and incomplete removal during wastewater treatment processes, TA has been increasingly detected in aquatic environments.^3^ The presence of this compound in surface waters raises environmental concerns since it may induce toxic effects in aquatic organisms, including oxidative stress and cellular damage. Furthermore, transformation processes occurring in natural waters, such as photochemical and oxidative reactions, may generate intermediate products with enhanced toxicity.^4^

The occurrence of TA in environmental systems highlights the importance of developing reliable analytical methods for monitoring. Several analytical approaches have been reported for the determination of TA in different matrices, including spectrophotometric,^5^ spectrofluorimetric,^6^ chromatographic,^7^ and electrophoretic techniques,^8^ and Fourier-transform infrared spectroscopy (FT-IR).^9^ Among them, chromatographic techniques combined with extraction procedures remain the most widely employed due to their excellent sensitivity and selectivity. Although these methods provide excellent sensitivity and selectivity, they generally rely on expensive instrumentation, labor-intensive sample preparation, and laboratory-based operation, which limits their applicability for rapid, on-site, and real-time environmental monitoring.

Considering these limitations, electrochemical techniques have emerged as attractive alternatives for the monitoring of pharmaceutical contaminants.^10–12^ These approaches offer several advantages, including high sensitivity, relatively low cost, rapid analysis, the possibility of miniaturization, and suitability for real-time monitoring. In particular, electrochemical sensors have received increasing attention for the determination of emerging contaminants in environmental matrices due to their simplicity, portability, and potential for in situ analysis.^13^ However, despite these advantages, to the best of our knowledge, only a limited number of electrochemical methods have been reported for TA determination,^1^ and the use of 3D-printed electrodes combined with plasma-activated surface modification for this purpose remains largely unexplored

Recent advances in additive manufacturing (AM), particularly the fused filament fabrication (FFF) technique, have increased the possibilities for fabricating electrochemical sensing platforms, offering advantages such as rapid prototyping, low cost, and high design flexibility. Most studies have relied on commercially available conductive filaments, whose exact composition is often not fully disclosed. However, some investigations have demonstrated that metallic impurities (e.g., Ti and Fe) in commercial filaments may significantly influence the electrochemical response of 3D-printed electrodes, which can affect analytical performance.^14,15^

In this way, custom-synthesized filaments have emerged as a promising route for the development of electrochemical sensors.^16,17^ Compared with commercial alternatives, these materials allow greater control over the type and loading of conductive fillers as well as the polymer matrix, while minimizing uncertainties associated with undisclosed additives and impurities that may affect electrochemical performance.^18^ The filament fabrication is based on two routes: the solvent-assisted route or the thermal route. The first utilizes organic solvents such as chloroform for the polymer dissolution, followed by the addition of conductive filler and a recrystallization step using ethanol.^16^ On the other hand, the thermal route comprises a melting step of the polymeric matrix, and subsequently it mixes the conductive material to form the composite.^16^ Although the solvent route often allows the incorporation of a higher percentage of conductive load, recent studies have focused on developing solvent-free processing routes capable of incorporating high loadings of conductive materials while improving the sustainability of filament fabrication.^19,20^

Despite the advantages associated with custom-made conductive filaments, 3D-printed electrodes often require postprinting activation to achieve optimal electrochemical performance. During the printing process, the insulating polymer matrix may partially cover the conductive filler particles, limiting the exposure of electroactive sites and hindering electron-transfer processes at the electrode surface.

To address this limitation, several activation approaches have been proposed, including mechanical polishing,^21^ solvent treatment,^22^ chemical oxidation,^23^ and electrochemical activation.^24^ In addition, laser-based methodologies have been reported as in blue-laser ablation,^25^ Nd:YAG (neodymium-doped yttrium aluminum garnet) laser^26^ and CO_2_ infrared laser.^27^ However, these methodologies still rely on cost-consuming instruments or environmentally aggressive reagents. In this scenario, there has been a demand for more environmentally amicable protocols.^28^

Among these protocols, plasma treatment has emerged as a suitable alternative because it promotes tailored surface modification without the use of hazardous chemical reagents or organic solvents in a wide variety of materials.^29,30^ Plasma is constituted by an ionized gas mix comprising ions, radicals, electrons, excited species, and neutral particles produced by the application of an energy source, such as an electric discharge or radiation.^31^ According to the gas temperature, plasma is categorized as thermal plasma (high-temperature particles) and nonthermal (cold) plasma (low-temperature particles). The gas source is an inert gas (e.g., Ne, Ar, O_2_) or air itself. Thermal plasma is not adequate for polymeric surface modifications because it might damage the material.^32^ However, nonthermal plasma can alter surface wettability, printability, adhesion, roughness, and remove polymeric layers by etching.^32^

Recently, Siqueira et al. showed the use of air plasma jet pens to activate 3D-printed electrodes made from commercial poly­(lactic acid) filament containing carbon black as conductive filler.^33,34^ These portable devices generate reactive plasma species directly from atmospheric air, enabling rapid and reagent-free surface activation without the need for external gases or chemical reagents.^35^ The reactive species produced during plasma generation can modify the electrode surface, promote partial removal of the insulating polymer matrix and increase the exposure of conductive carbon domains.^34^ Therefore, atmospheric air plasma pen systems offer a simple, rapid, and reagent-free route for the activation of 3D-printed electrodes, making them especially attractive for sustainable fabrication of electrochemical sensing platforms.

Herein, this work aims to develop a potentially cost-effective electrochemical platform for the determination of TA based on a 3D-printed carbon black/polylactic acid (CB/PLA) electrode. The conductive filament was produced in-house through a solvent-free thermal mixing process, offering a potentially greener and readily adaptable route for electrode fabrication. Furthermore, the electrochemical performance of the CB/PLA electrode was significantly improved through a rapid and reagent-free surface activation using an atmospheric air plasma jet pen. The resulting CB/PLA-PT electrode was successfully applied to the voltammetric determination of TA in environmental water samples. To the best of our knowledge, this study is the first to report the use of a 3D-printed electrochemical sensor for TA detection, emphasizing the innovative combination of additive manufacturing and plasma-assisted surface activation for environmental monitoring applications.

## 2 Experimental Section

### 2.1 Reagents, Solutions, and Samples

All aqueous solutions were freshly prepared using ultrapure water with a resistivity of 18.2 MΩ cm at 25 °C, obtained from a Direct 8 system (Millipore, USA). All reagents used were of analytical grade and were used without further purification. Tolfenamic acid (TA, 99%w/w), flunixim (FLU, 99%w/w), gentamicin (GNT, 99%w/w), ivermectin (IVT, 99%w/w), uric acid (UA, 99%w/w), and sulfamethoxazole (SFT, 99%w/w) analytical standards were purchased from Sigma-Aldrich (USA). Boric acid (99.5%w/w) and ethanol (99.5%w/w) were purchased from Vetec Química (Brazil), glacial acetic acid (99%w/w), sodium hydroxide (99%w/w), and orthophosphoric acid (85%w/w) were purchased from Merck (Germany). Potassium chloride (99%w/w) was acquired from from Vetec (Brazil) and potassium ferricyanide (99%w/w) was purchased from Grupo Química (Brazil). CB particles (VXC72 type) were acquired from Cabot (São Paulo, Brazil), while PLA pellets were purchased from 3D LAB (Minas Gerais, Brazil), and castor oil was purchased from local suppliers (Rio de Janeiro, Brazil).

Britton-Robinson buffer solution (BR) (0.1 mol L^–1^) was prepared as the supporting electrolyte. The pH values of this solution were adjusted within the range of 5.0–8.0 using a saturated NaOH solution. Stock solutions (1 mmol L^–1^) of TA, FLU, and GNT solutions were prepared in ethanol. A 1 mmol L^–1^ SFT was prepared in deionized water, while a 5 mmol L^–1^ UA solution was prepared in BR buffer (pH = 2.0). The potassium ferricyanide solution (1 mmol L^–1^) was prepared in 0.1 mol L^–1^ KCl. Tap and river water samples were analyzed in this study. The tap water samples were collected directly from the laboratory, while river water samples were collected from Porto Firme—Minas Gerais, Brazil (20°39′44″S 43°5′1″W). The samples were diluted 2-fold in BR buffer (pH = 7.0) and spiked at two different concentration levels before analysis.

### 2.2 Instrument and Apparatus

The electrochemical measurements were performed using a PGSTAT204 potentiostat (Metrohm, The Netherlands) controlled by NOVA software (version 2.1). Differential pulse voltammetry (DPV) data were processed using the baseline correction tool available in NOVA software. Baseline subtraction was carried out using the moving average mode with a window size of 1, and the resulting baseline-corrected voltammograms were used for peak current measurements.

Scanning electron microscopy (SEM) images were obtained in a FEI SEM/FIB DualBeam Nova NanoLab 600 microscope (Hillsboro, USA) using 2.0 kV and 0.21 nA parameters. Attenuated total reflectance Fourier-transform infrared spectroscopy (ATR-FTIR) spectra were obtained in a Bruker INVENIO R spectrometer (Billerica, USA) equipped with a Platinum ATR accessory containing a single-reflection diamond crystal. The measurements were obtained in room temperature from 4000 to 600 cm^–1^. Raman spectra were measured in a Witec (Ulm, Germany) Alpha300 AR confocal spectrometer using a 532 nm laser excitation wavelength in the spectral range from 200 and 3800 cm^–1^. Static water contact-angle measurements were performed using the sessile-drop method. A 5 μL water droplet was deposited at three different locations on the surface of the same electrode (n = 3). Approximately 1 min after each droplet deposition, the electrode surface was photographed using a smartphone camera positioned horizontally at the level of the surface to minimize perspective distortion. The contact angles were subsequently estimated by image analysis using GeoGebra Classic software, and the results were expressed as mean ± standard deviation.

### 2.3 Conductive CB/PLA Filament Production

The conductive filaments were prepared using CB, PLA, and castor oil, following a mixing thermal route previously reported in the literature.^36^ Initially, a total mass of 30.0 g comprising PLA and CB was weighed, maintaining a mass ratio of 20:80%w/w (CB:PLA).

Subsequently, castor oil was added at 15% w/w relative to the total mass of the CB/PLA blend, corresponding to 4.5 g. It was incorporated as a biobased plasticizer and binder to improve the flexibility and processability of the composite, facilitating filament extrusion and subsequent 3D printing. The plasticizer content and the general thermal mixing route were maintained from the methodology reported by Silva et al.,^36^ while the soybean oil used in the original formulation was replaced with castor oil in the present study. This substitution was based on recent reports demonstrating the suitability of castor oil as a plasticizer for conductive PLA-based filaments and additively manufactured electrochemical electrodes.^37^

The components were mixed and heated on a hot plate at approximately 180 °C, with continuous manual stirring using a glass rod to promote melting and uniform dispersion of the conductive particles. After incorporation of CB into the thermoplastic matrix, the composite material was transferred onto a Petri dish, where it was spread into a thin layer and allowed to cool to room temperature. The CB/PLA composite material was grinded using an industrial double-shaft shredder (Filmaq 3D). The grinded composite was extruded using an extrusion machine operated at 200 °C (Filmaq 3D). The electrodes were printed using a Bambu Lab A1 3D-printer (Bambu Lab, China) (Scheme 1A).

**Scheme 1:**
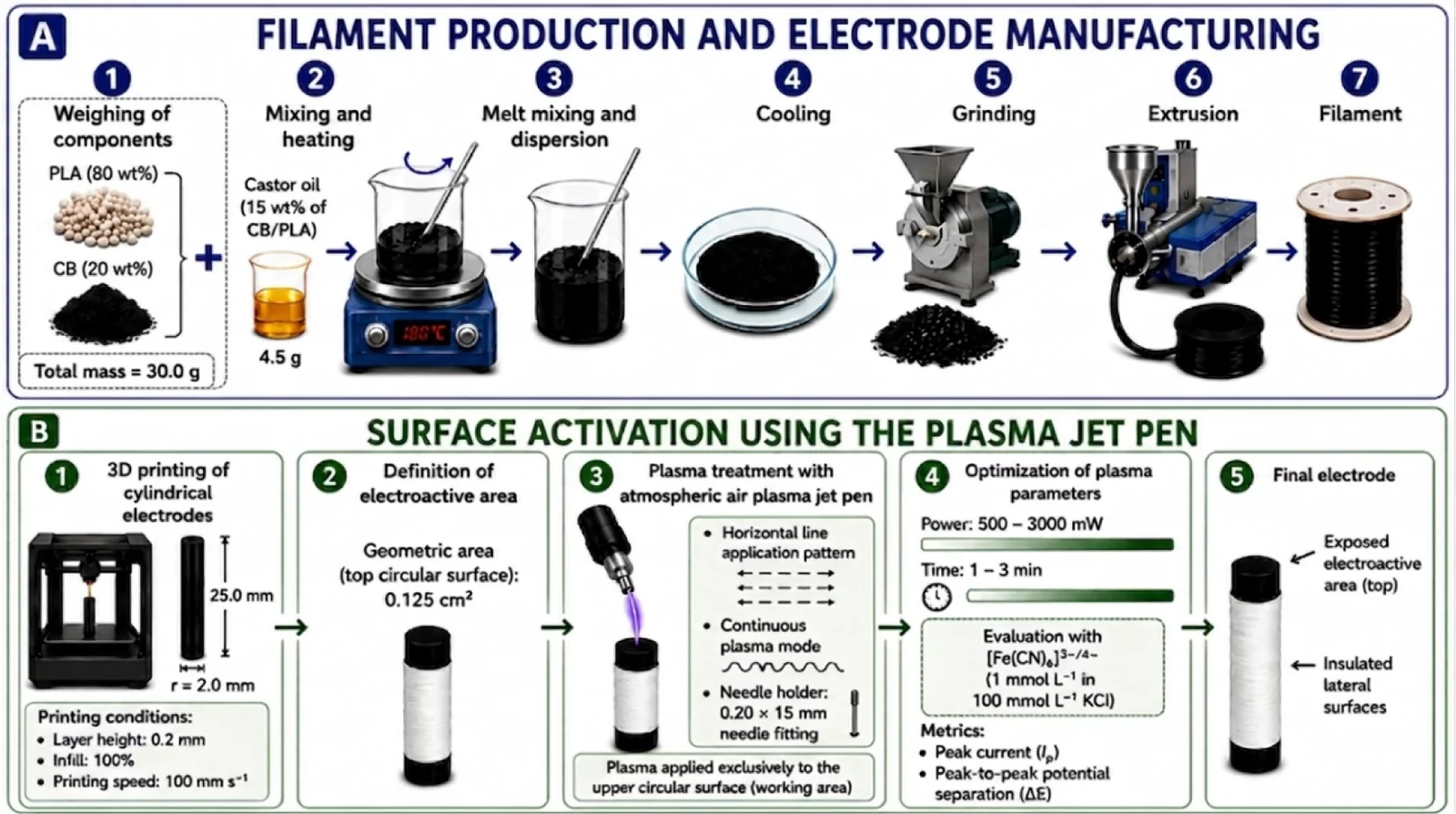
(A) Representation of the Filament Production and Electrode Manufacturing; (B) Representation of the Surface Activation Using the Plasma Jet Pen^a^

a The illustrative elements were generated using ChatGPT and subsequently edited by the authors.

Therefore, cylindrical working electrodes with dimensions of 25.0 mm in length and a radius of 2.0 mm were printed under the following printing conditions: conductive CB/PLA filament diameter of 1.75 mm, nozzle of 0.4 mm operating at 220 °C with a bed temperature of 60 °C, layer height of 0.2 mm, 100% infill, and a printing speed of 100 mm s^–1^ (Figure S1). Thus, the electroactive area used corresponded to the circular top surface of the electrode, with a geometric area of 0.125 cm^2^ (Figure S1). This area was defined by partially insulating the lateral surfaces of the cylinder with nail polish and Teflon tape. Electrical contact with the potentiostat was established directly by placing the potentiostat cable in contact with the printed CB/PLA material at the end opposite to the end used as the electrode surface. Scheme 1A summarizes the production of CB/PLA composite filament used in this work.

### 2.4 Treatment of the 3D Electrodes with the Atmospheric Air Plasma Jet Pen

The electrodes underwent surface activation using an atmospheric air plasma jet pen PLASMAX-EHF 2204 (KLD Biosistemas, Brazil). It was used a methodology adapted from the literature.^34^ Therefore, the horizontal line application pattern, continuous plasma mode, and the needle holder configuration (0.20 × 15 mm needle fitting) were adopted. Plasma treatment was applied exclusively to the working area of the electrode, corresponding to the upper circular surface of the cylinder. The plasma tip was positioned 1 mm above the electrode surface, and the treatment was performed manually with horizontal movements across the electrode surface for a fixed treatment time. Since the activation was performed manually, the number of passes and the movement rate were not specifically controlled; instead, the treatment time was used as the controlled parameter. Immediately after plasma treatment, the activated electrode was transferred to the electrochemical cell, with approximately 1 min between plasma activation and the electrochemical measurements. To evaluate the effect of plasma conditions on surface activation, the applied power varied from 500 to 3000 mW, while the treatment time ranged from 1 to 3 min. The impact of these parameters on the electrochemical performance was assessed using 1 mmol L^–1^ potassium ferricyanide in 0.1 mol L^–1^ KCl as a redox probe, considering the peak current (*I*p) and peak-to-peak potential separation (Δ*E*). The process is illustrated in Scheme 1B.

### 2.5 Electrochemical Measurements

A 10 mL glass beaker was used as an electrochemical cell. The conventional counter (Pt wire) and reference (Ag|AgCl|KCl_(sat.)_) electrodes were used for electrochemical measurements. Cylindrical working electrodes were printed and subsequently activated with the air plasma jet pen. The influence of pH on the TA oxidation (5 μmol L^–1^) was investigated by differential pulse voltammetry (DPV) using BR buffer over the pH range of 5.0–8.0.

Additionally, cyclic voltammetry (CV) measurements were performed between 0.0 and 1.2 V (vs Ag|AgCl|KCl_(sat.)_) in a solution containing 100 μmol L^–1^ TA prepared in BR buffer (pH 7.0), with scan rates varying from 10 to 225 mV s^–1^. Electrochemical impedance spectroscopy (EIS) experiments were carried out in a solution of 1 mmol L^–1^ K_3_[Fe­(CN)_6_] and 0.1 mol L^–1^ KCl as the supporting electrolyte. The frequency range was set from 100 kHz to 0.01 Hz, with an amplitude of 10 mV and 10 data points per frequency decade.

## 3 Results and Discussion

### 3.1 Morphological Characterization of the CB/PLA-PT Electrode

First, SEM analysis was performed to evaluate the morphological changes induced by the plasma pen activation treatment on the surface of the 3D-printed electrode. Figures 1A and S2A reveal a homogeneous and uniform surface of the CB/PLA electrode. Before the treatment, the electrode surface exhibited spherical CB particles dispersed and partially CB embedded within the smooth PLA matrix. The plasma activation treatment is expected to preferentially remove the PLA-rich surface layer, thereby exposing a greater fraction of the conductive CB particles and improving the electrochemical accessibility of the electrode surface.^34,38^ This effect is clearly evidenced in the SEM images of the CB/PLA-PT electrode (Figures 1B and S2B), where the plasma-chemical PLA etching leads to the formation of sponge-like structures, with the appearance of numerous cavities and microholes distributed across the electrode surface. Both CB/PLA and CB/PLA-PT electrode surfaces were also characterized by FTIR (Figure 1C) and Raman (Figure 1D) spectroscopies.

**Figure 1:**
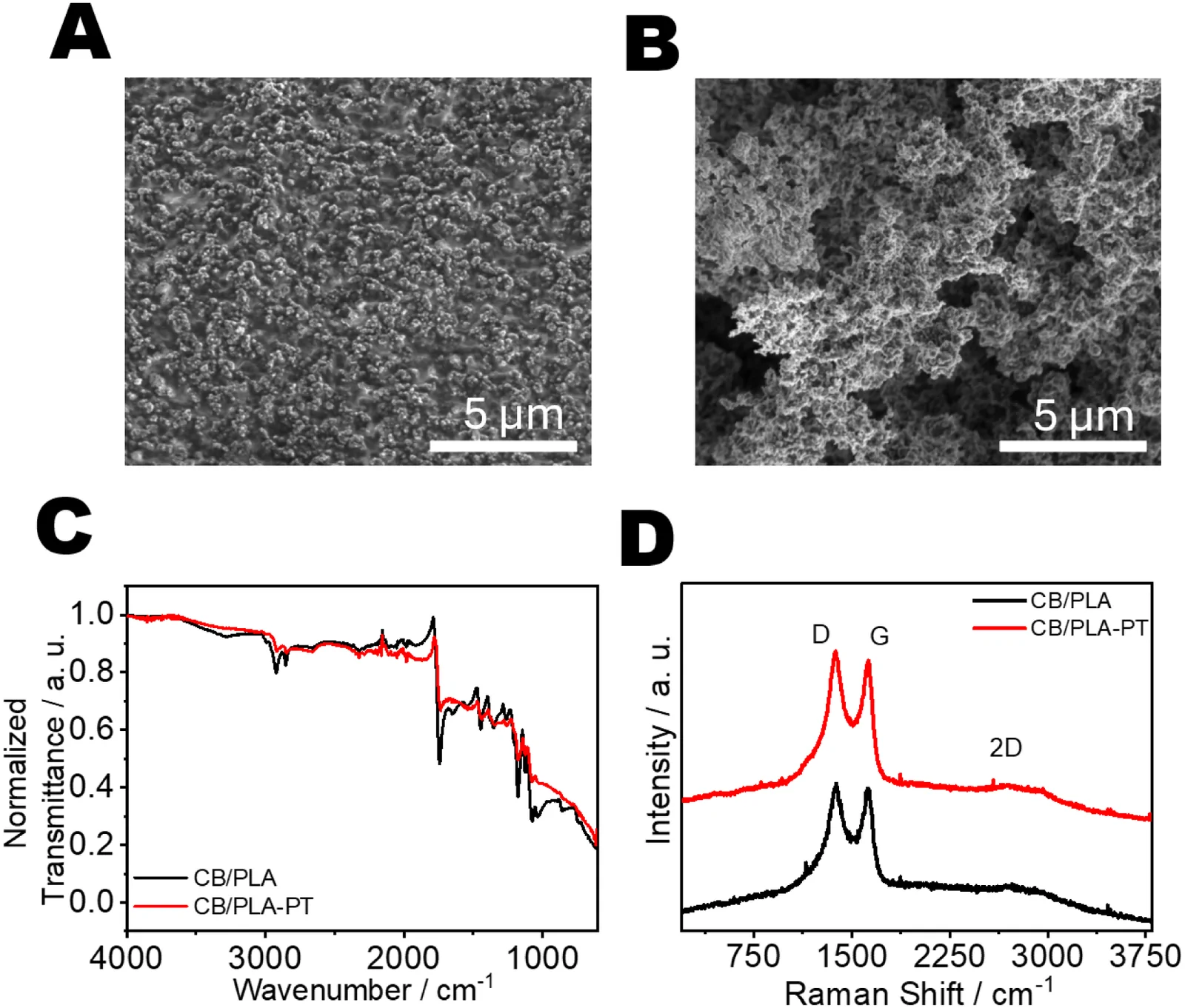
(A) SEM images acquired for CB/PLA electrodes with HFW 17.1 μm. (B) SEM images obtained for the CB/PLA-PT electrodes with HFW 17.1 μm. (C) ATR-FTIR and (D) Raman spectra of CB/PLA (black line) and CB/PLA-PT (red line) electrodes.

FTIR spectra show the vibrational bands of the polymeric PLA matrix, such as the strong absorbance band at 1733 cm^–1^, which is associated with carbonyl (C=O) stretching, bands associated with vibrations from the methyl groups (−CH_3_) appear at 2911 and 2846 cm^–1^ for C–H stretching and at 1448 cm^–1^ for the bending mode. In addition, the bands between 1177 and 1027 cm^–1^ are associated with C–O stretching from the ester group.^39,40^ For the CB/PLA-PT the bands were the same, but a noticeable decrease in the intensity of the PLA bands was observed, particularly those associated with the methyl (−CH_3_) and carbonyl (C=O) groups. These results indicate a partial removal of the polymeric matrix after plasma treatment and agreed with the SEM images, which revealed increased exposure of the CB particles to the electrode surface. Such surface modification favors electron-transfer processes by increasing the accessibility of electroactive sites.

Raman spectroscopy of both electrodes (Figure 1D) revealed the carbon-based materials characteristic D and G bands at 1380 and 1621 cm^–1^, respectively, together with a second-order 2D weak broad band at 2688 cm^–1^. The G band corresponds to the in-plane C–C bonds stretching attributed to E_2g_ vibration mode,^41^ while the D band (A1g) is associated to defects in the carbon structure.^42,43^ Carbon materials, like CB, the D and G-bands appear with similar intensities and wide bands widths.^44^ The degree of the structural disorder can be quantified by the I_D_/I_G_ ratio, which was 1.02 before the plasma activation treatment and slightly increased to 1.03 after activation. This minimal change indicates that the plasma treatment did not substantially alter the structure of the CB, supporting the interpretation that the main effect of the treatment occurs at the polymer-rich surface, increasing CB exposure through partial removal of the PLA insulating phase. This behavior is consistent with previous studies employing plasma pen activation on 3D-printed carbon-based electrodes,^34,45^ which also reported negligible changes in Raman spectra after treatment, suggesting that plasma activation primarily affects the polymeric phase rather than the carbon framework.

Water contact-angle measurements were performed to evaluate the changes in surface wettability induced by plasma treatment (Figure S3A–B). The contact angle decreased from 56.1 ± 5.3° for the CB/PLA electrode to 41.3 ± 3.9° for the CB/PLA-PT electrode (n = 3), corresponding to a 26.4% reduction. This decrease indicates enhanced surface wettability after plasma treatment, potentially associated with increased surface polarity. Such behavior may result from changes in surface chemistry, including the possible formation or incorporation of oxygen-containing functional groups, such as hydroxyl, carbonyl, and carboxyl species. This interpretation is consistent with previous studies reporting oxygen-containing functionalities on plasma-treated carbon surfaces.^34,46^ If present, these polar functionalities could enhance the interaction between the electrolyte and the electrode surface, thereby contributing to the improved electrochemical response. Therefore, the contact-angle results indicate increased wettability and are consistent with possible changes in the surface polarity induced by plasma treatment.

### 3.2 Optimization of Plasma Pen Treatment on 3D-Printed CB/PLA Surface

Initially, the plasma activation conditions were optimized by evaluating the electrochemical response of 1.0 mmol L^–1^ potassium ferricyanide in 0.10 mol L^–1^ KCl after treatment under different power (500–3000 mW) and exposure time conditions (1–3 min). Considering the quasi-reversible behavior of the ferri/ferrocyanide redox couple on carbon-based composite electrodes, the optimization was based on the peak-to-peak separation (Δ*E*), the anodic-to-cathodic peak current ratio (*I*_a_/*I*_c_), and the peak current values. The corresponding cyclic voltammograms are presented in Figure S4A–B, while the obtained electrochemical parameters are summarized in Figure S4C–D. As can be seen, an increase in applied power, using a fixed time of 1 min, resulted in a clear enhancement of the peak current, accompanied by a decrease in peak-to-peak separation (Δ*E*) at higher power values. Based on these findings, 3000 mW was selected as the optimal power for surface treatment. It is important to mention that higher power values were not evaluated because 3000 mW corresponds to the maximum output power of the plasma pen system.

The effect of treatment time was subsequently investigated using the selected power value. Among the evaluated conditions, a treatment time of 2 min resulted in the highest peak currents and the lowest Δ*E* values, indicating the most reversible electrochemical behavior. Therefore, 3000 mW and 2 min were established as the optimal parameters for surface activation.

To further evaluate the effect of plasma activation on the electrochemical behavior of the CB/PLA electrode, cyclic voltammograms of the ferri/ferrocyanide redox probe were recorded before and after treatment (Figure 2A). As observed, the CB/PLA-PT electrode exhibited an improvement in electrochemical performance toward the potassium ferricyanide (1 mmol L^–1^ in 0.10 mol L^–1^ KCl) redox probe. The anodic peak current increased from 12.6 to 50.8 μA, corresponding to an enhancement of approximately 400%, while the Δ*E* decreased significantly from 699 to 261 mV. These results indicate a considerable improvement in the reversibility of the electrochemical process and faster electron-transfer kinetics after plasma treatment. In addition, the |*I*_a_/*I*_c_| ratio remained close to unity for both electrodes, suggesting preservation of the redox response symmetry despite the pronounced increase in current intensity.^34^

**Figure 2:**
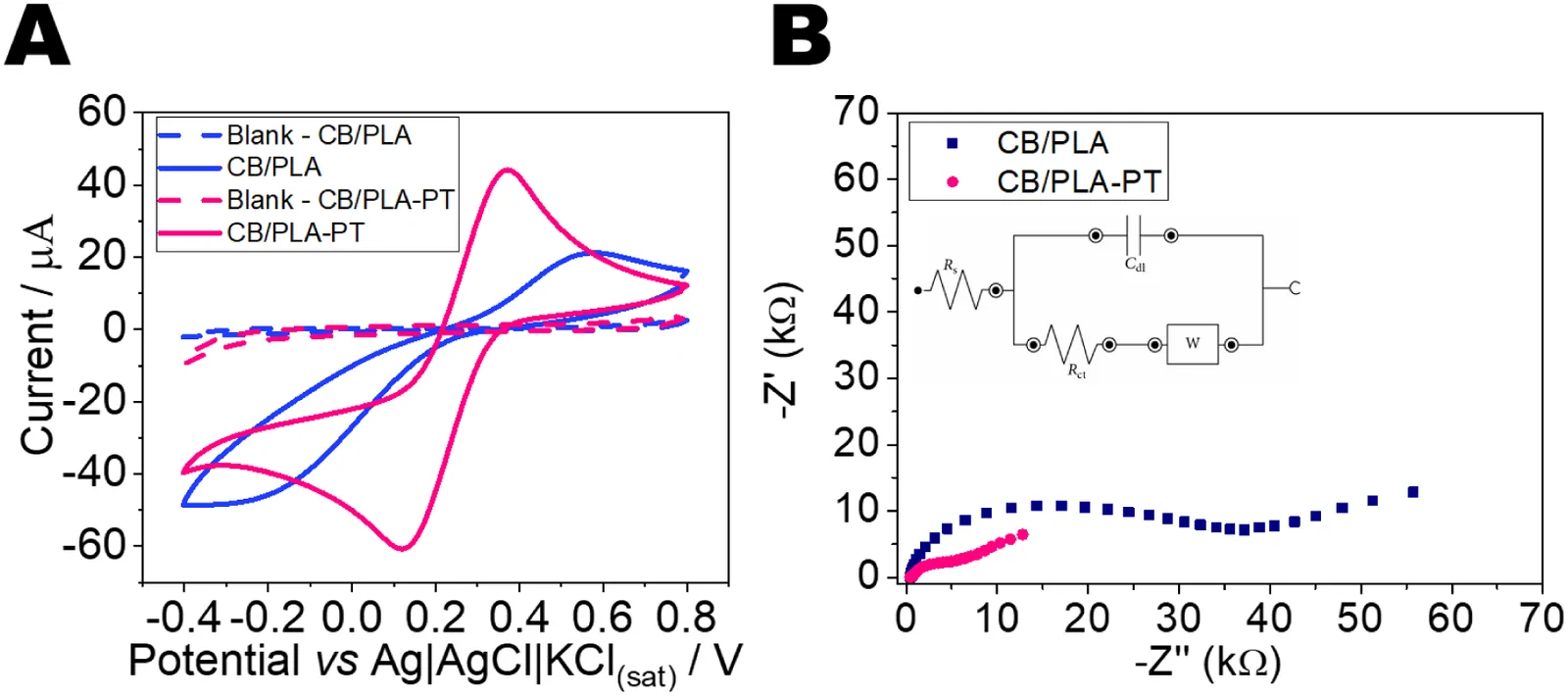
(A) CV recorded in 1 mmol L^–1^ K_3_[Fe­(CN)_6_] containing 0.1 mol L^–1^ KCl at CB/PLA (blue line) and CB/PLA-PT (pink line) electrodes. The dashed lines represent the blanks for each measurement. Experimental conditions: scan rate of 50 mV s^–1^ and step potential of 5 mV. (B) EIS of CB/PLA (blue dots) (before plasma activation) and CB/PLA-PT (pink dots) (after plasma activation) obtained in a solution of 1 mmol L^–1^ K_3_[Fe­(CN)_6_] containing 0.1 mol L^–1^ KCl. EIS conditions: frequency range from 100 kHz to 0.01 Hz, amplitude of 10 mV, and open-circuit potential of +0.365 V (vs Ag|AgCl|KCl_(sat.)_). The impedance data were fitted using a Randles equivalent circuit model.

The EIS results also corroborate the enhancement in interfacial charge-transfer properties. The charge-transfer resistance (*R*_ct_), obtained from Nyquist plots (Figure 2B), decreased from 33.4 kΩ for the CB/PLA electrode to 7.56 kΩ for the CB/PLA-PT electrode, representing a ∼4.4-fold reduction. The decrease in R_ct_ indicates a faster charge-transfer process at the CB/PLA-PT electrode surface, consistent with the previous voltammetric results.

The double-layer capacitance (C_dl_) values were estimated from measurements obtained by cyclic voltammetry in a nonfaradaic region (Figure S5A–D) using a previously reported methodology.^47^ Cyclic voltammograms were recorded in 3.0 mol L^–1^ KCl solution within a potential window from 0.0 to 1.2 V at scan rates ranging from 10 to 125 mV s^–1^. The capacitive current density variation (ΔJ) measured at 0.3 V was plotted as a function of scan rate, and the slope of the linear regression corresponded to the C_dl._ The C_dl_ value increased from 10.1 μF cm^–2^ for the CB/PLA electrode to 45.7 μF cm^–2^ for the CB/PLA-PT electrode, suggesting an increase in the electrochemically accessible interfacial area. Table 1 summarizes the electrochemical characterization results for the untreated and treated electrodes.

**Table 1:** Data from the Electrochemical Characterization of the CB/PLA and CB/PLA-PT Electrodes (n = 3)

Electrode	*I*_a_/μA	I_c_/μA	|*I*_a_/*I*_c_|	Δ*E*/mV	*R*_ct_/kΩ	C_dl_/μF cm^–2^
CB/PLA	12.6 ± 0.3	11.0 ± 0.5	1.14 ± 0.05	699 ± 10	33.4 ± 1.5	10.1 ± 0.15
CB/PLA-PT	50.8 ± 1.5	–47.5 ± 0.8	1.06 ± 0.03	261 ± 5	7.56 ± 0.65	45.7 ± 0.95

Taken together, the morphological, spectroscopic, wettability, and electrochemical results provide a consistent mechanistic explanation for the enhanced performance of the CB/PLA-PT electrode. The partial removal of the PLA-rich surface layer, evidenced by the SEM morphology and the decrease in the characteristic PLA bands in the FTIR spectra, increases the exposure and accessibility of the conductive CB phase without substantially altering its carbon structure, as supported by the negligible change in the Raman I_D_/I_G_ ratio. In parallel, the decrease in the water contact angle indicates improved surface hydrophilicity, which facilitates electrolyte wetting and promotes better contact between the solution and the exposed conductive domains. These combined surface modifications increase the accessibility of electroactive sites and facilitate interfacial charge transfer, as evidenced by the approximately 4.4-fold decrease in R_ct_ and the marked increase in C_dl_. Consequently, the faster electron-transfer kinetics are reflected in the higher ferri/ferrocyanide peak currents and lower ΔE observed after plasma activation. The resulting more accessible, conductive, and hydrophilic electrode interface is therefore expected to facilitate the electron-transfer processes associated with TA oxidation, ultimately contributing to the enhanced analytical response observed for the CB/PLA-PT sensor.

### 3.3 Electrochemical Behavior of TA on 3D-Printed Electrochemical Electrodes

Subsequently, an exploratory DPV experiment was performed using 5 μmol L^–1^ TA in 0.10 mol L^–1^ BR buffer at pH 6.0, selected as a near-neutral starting condition within the pH range previously investigated for the electrochemical oxidation of TA in BR buffer,^48^ to assess the effect of plasma treatment on the analytical response (Figure 3A). The CB/PLA electrode (orange line) exhibited an oxidation peak current of 0.31 μA at approximately +0.76 V (vs. Ag|AgCl|KCl_(sat.)_), whereas the CB/PLA-PT electrode (purple line) produced a peak current of 0.46 μA at +0.65 V.

**Figure 3:**
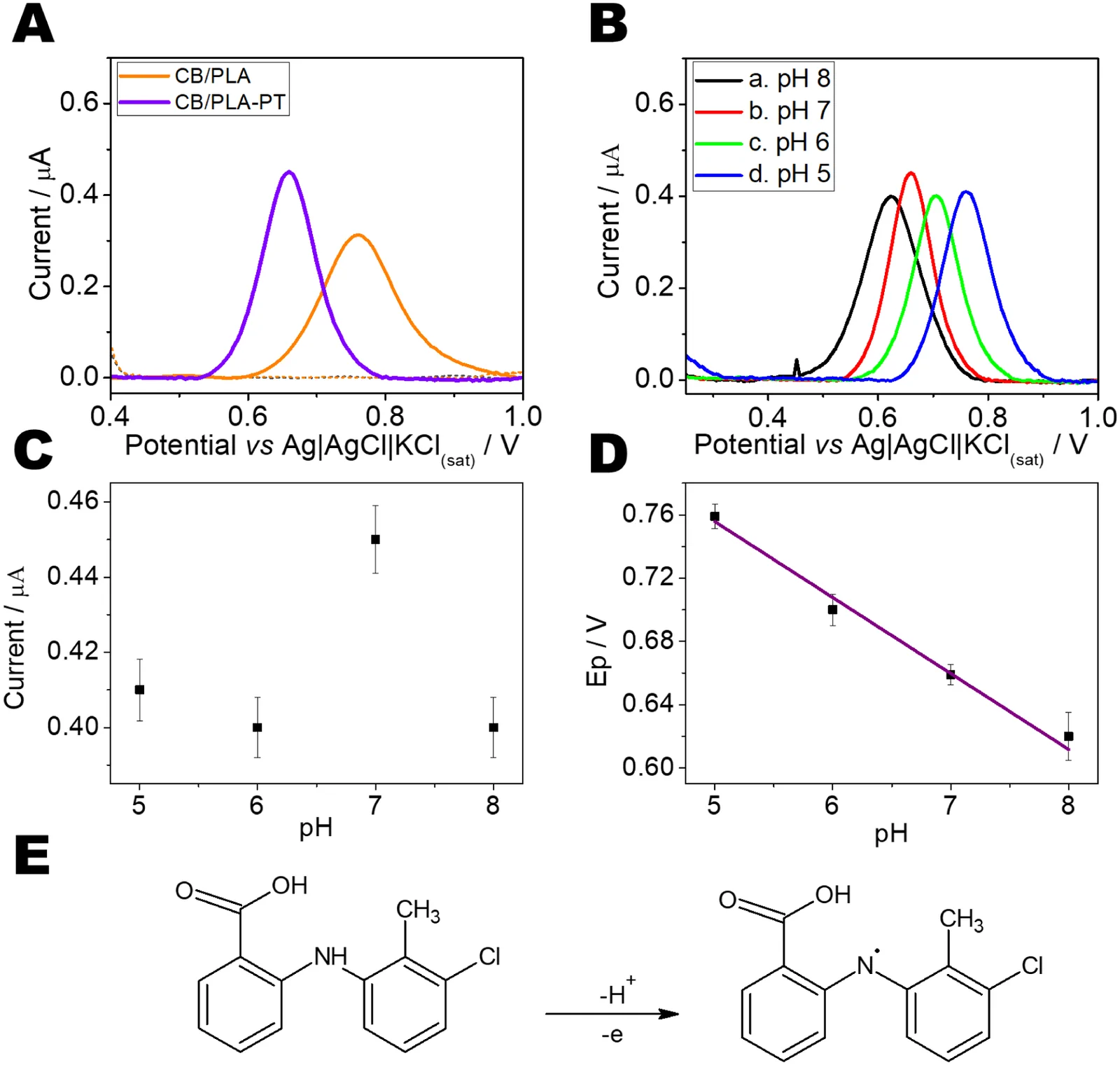
(A) Exploratory comparison of the DPV responses of TA (5.0 μmol L^–1^) in 0.1 mol L^–1^ BR buffer at pH 6.0 using CB/PLA (orange line) and CB/PLA-PT (purple line) electrodes. (B) DPV responses of TA (5.0 μmol L^–1^) in BR buffer at different pH values (5.0–8.0) (*n* = 3). (C) Dependence of the oxidation peak current (I_p_) on pH. (D) Dependence of the oxidation peak potential (Ep) on pH. (E) Proposed oxidation mechanism of TA at the CB/PLA-PT electrode. DPV conditions: potential step, 5 mV; pulse amplitude, 25 mV; modulation time, 50 ms; scan rate, 10 mV s^–1^.

Thus, plasma treatment resulted in an approximately 1.5-fold increase in peak current and a 110 mV shift toward a less positive oxidation potential. These changes indicate that TA oxidation was facilitated at the plasma-treated electrode surface and are consistent with a lower overpotential and improved interfacial electron transfer. Such behavior may be associated with the surface modifications induced by plasma treatment.^20^ The observed morphological changes may increase the electrochemically accessible interfacial area and promote more favorable electron-transfer kinetics. Therefore, the improved voltammetric response observed for TA may result from the combined effects of greater exposure of carbon active sites, increased electrochemically accessible interfacial area, and possible changes in surface polarity, including the possible formation of oxygen-containing functionalities during plasma treatment. Together, these effects may contribute to the enhanced electrochemical response of the electrode toward TA oxidation.

DPV measurements were also explored to investigate the impact of pH on the electrochemical response of TA. For this purpose, additional voltammetric analyses were performed in BR buffer over the pH range 5.0–8.0 using a 5.0 μmol L^–1^ TA solution. This pH range was selected considering the p*K*_a_ value of TA (∼4.0), since under these conditions the molecule is predominantly in its deprotonated form. As shown in Figure 3B–D, the anodic peak potential shifted toward less positive values with increasing pH, while the oxidation current increased up to pH 7.0. Therefore, pH 7.0 was selected for subsequent experiments, as it provided the highest analytical response without a significant change in peak potential compared to a relatively acidic medium, such as pH 6.0. A linear dependence between peak potential and pH was observed for TA oxidation (see Figure 3D). The slope (−48 mV pH^–1^) is close to the theoretical Nernstian value expected for reactions involving the same number of protons and electrons.^49^ Although the slope slightly deviates from the ideal value of 59 mV pH^–1^, this behavior is consistent with the irreversible behavior of the oxidation process.

Previous electrochemical studies on fenamate nonsteroidal anti-inflammatory drugs, such as TA and analogues, have reported irreversible oxidation behavior attributed to the diphenylamine structure common to this class of compounds^48^ (Figure 3E). The observed pH dependence supports a proton-coupled electron transfer (PCET) mechanism, in which electron and proton transfers are involved in the oxidation reaction.^50^ Overall, the electrooxidation of TA at the CB/PLA-PT electrode surface is consistent with a PCET process. The pH dependence of the peak potential suggests the involvement of the same number of protons and electrons in the oxidation mechanism, likely associated with the oxidation of the diphenylamine moiety of the molecule.

Additionally, CV experiments were carried out using 100 μmol L^–1^ TA BR buffer (pH 7.0) at scan rates ranging from 10 to 225 mV s^–1^ using the CB/PLA-PT electrode (Figure S6A). The anodic peak current increased progressively with increasing scan rate, and a linear relationship between peak current (*I*_p_) and the square root of the scan rate (*v* ^1/2^) was observed (Figure S6B), indicating that the electrooxidation process is predominantly diffusion-controlled. Additionally, a log–log plot of *I*_p_ versus scan rate was constructed (Figure S6C). The linear regression yielded a slope of 0.49 (R^2^ = 0.997), which is very close to the theoretical value of 0.5 expected for diffusion-controlled electrochemical processes.^49^

### 3.4 Analytical Performance of the 3D-Printed CB/PLA-PT for TA Determination

#### 3.4.1 Instrumental DPV Parameters Optimization

The influence of the instrumental parameters of DPV on the analytical response was evaluated in 0.1 mol L^–1^ BR buffer (pH 7) containing 5 μmol L^–1^ TA, and the corresponding voltammograms are presented in Figure S7. The instrumental parameters were evaluated individually using a univariate approach, following the sequence described below. The optimal conditions were selected by seeking a compromise between the intensity of the electrochemical response and the definition of the voltammetric peak of TA.

The effect of the step potential was investigated (Figure S7A–B), and a progressive increase in the oxidation current was observed over the range of 1 to 5 mV. At 7 mV, however, a decrease in the current was observed, probably due to poorer definition of the voltammetric signal. Thus, a step potential of 5 mV was selected for subsequent analyses.

Regarding the pulse amplitude (Figure S7C–D), an increase in the oxidation current was observed as the amplitude increased from 25 to 75 mV, indicating an enhancement in signal intensity. However, higher amplitudes resulted in a decrease in the current, which may be associated with distortion of the voltammetric signal and a consequent loss of analytical performance. Therefore, 75 mV was selected as the optimum value. The modulation time was kept constant at 50 ms throughout the optimization study.

Finally, the optimum values for the DPV instrumental parameters were a step potential of 5 mV, pulse amplitude of 75 mV, and modulation time of 50 ms, using a scan rate of 5 mV s^–1^. Within the potential range from 0.45 to 0.80 V, which was sufficient to promote TA oxidation, the analysis could be completed in only 35 s.

#### 3.4.2 Assessment of Analytical Metrics and Application to Water Environmental Samples

After optimizing the instrumental conditions, DPV analyses (Figure 4A) were performed to establish the linear working range of the proposed CB/PLA-PT electrode. A linear calibration curve was obtained for TA in the concentration range from 1.91 to 20.1 μmol L^–1^ in BR buffer (pH 7.0) (Figure 4B). The calibration curve was constructed from three independent calibration curves, with three replicate measurements at each concentration level. TA concentrations were obtained by successive additions of a 1 mmol L^–1^ ethanolic TA stock solution directly into the electrochemical cell. Consequently, the ethanol content increased progressively from approximately 0.19 to 1.81% (v/v) over the investigated calibration range.

1 $$I_{p}\left(\mathrm{\mu A}\right)=\left(0.134\pm 0.002\right)\left({\mathrm{\mu mol}L}^{-1}\right)\left[\mathrm{TA}\right]+\left(0.027\pm 0.024\right)$$

**Figure 4:**
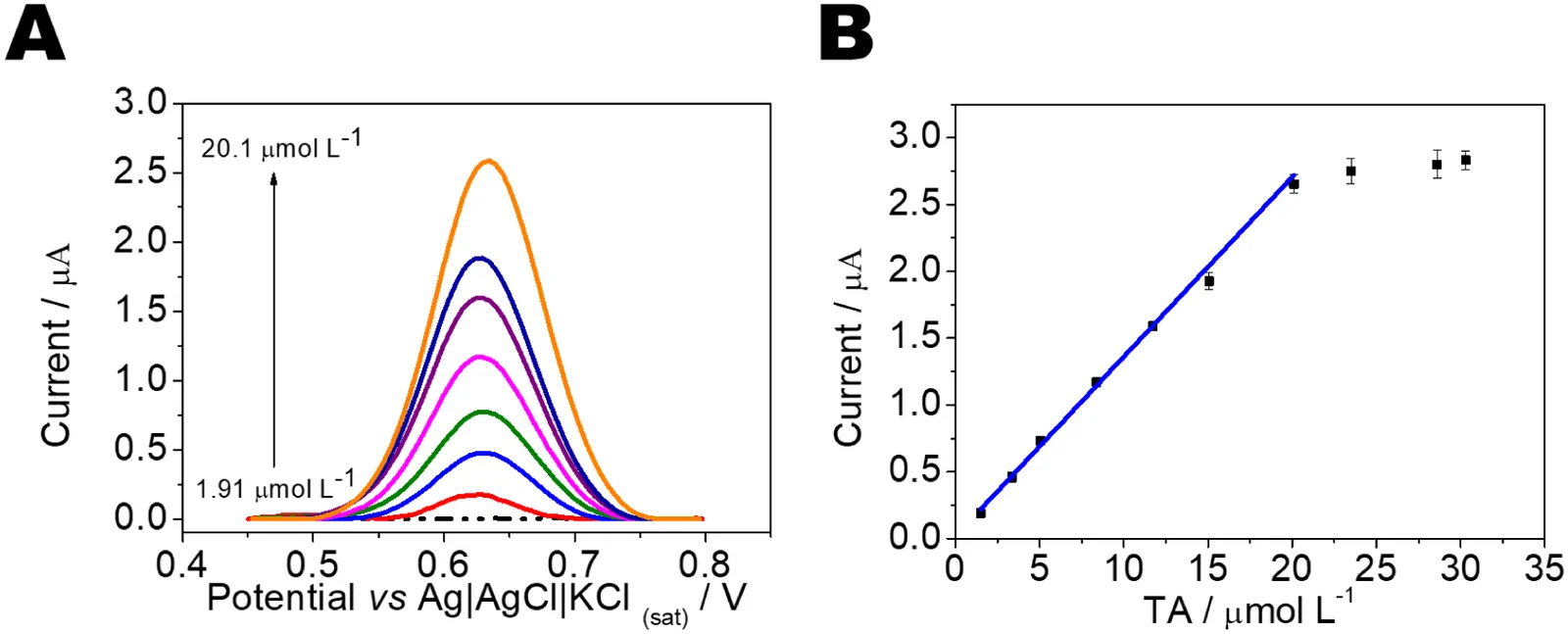
(A) DPV measurements recorded using increasing TA concentrations ranging from 1.91 to 20.1 μmol L^–1^ in 0.1 mol L^–1^ BR buffer (pH 7.0) upon CB/PLA-PT electrode (*n* = 3). (B) Plot of the linear relationship between TA concentration and the peak current. DPV conditions: step potential: 5 mV; amplitude: 75 mV; modulation time: 50 ms.

The regression coefficient was R^2^ = 0.993. The slope was 0.134 μA mol L^–1^, and the uncertainties associated with the slope and intercept represent the standard errors of the respective regression coefficients. The corresponding values were 0.134 ± 0.002 and 0.027 ± 0.024, respectively. Concentration levels above 20.1 μmol L^–1^ were also evaluated to identify the onset of deviation from linearity. The upper limit of the linear range was defined as 20.1 μmol L^–1^ because it was the highest concentration that maintained a linear analytical response. Higher concentrations showed evidence of surface saturation and were therefore excluded from the linear regression.

The analytical sensitivity was 0.134 μA L μmol^–1^, and the limits of detection (LOD) and of quantification (LOQ) were estimated according to IUPAC recommendations using the standard error of the intercept (s) and the slope of the analytical curve (A), where LOD = 3s/A and LOQ = 10s/A.^51^ The obtained LOD and LOQ values were 0.54 μmol L^–1^ and 1.80 μmol L^–1^, respectively.

The repeatability using the same sensor was assessed by performing nine consecutive measurements of a 20.1 μmol L^–1^ TA solution under identical experimental conditions (Figure S8A). Between measurements, no polishing, cleaning, or reactivation step was performed on the electrode surface. The electrode remained immersed in the same solution, which was only gently stirred for 10 s between measurements. This protocol was adopted to evaluate whether electrode fouling would compromise the sensor’s analytical performance under consecutive measurements, particularly at this higher TA concentration, where fouling effects arising from the irreversible oxidation of TA would be expected to be more pronounced. The peak currents obtained across the nine measurements did not display a systematic decay trend, and the relative standard deviation (RSD) value of 3.8% was achieved, demonstrating good precision and confirming that fouling did not significantly affect the repeatability of the CB/PLA-PT sensor under these conditions, without the need for surface regeneration between measurements.

Also, the interelectrode precision of the proposed sensor was evaluated using three independently fabricated electrodes in the determination of 5.0 μmol L^–1^ TA under the same experimental conditions (Figure S8B). The resulting RSD of 5.7% indicates satisfactory interelectrode precision and consistency of the fabrication process.

The selectivity of the proposed sensor was evaluated by investigating the influence of species likely to be present in environmental waters, including flunixin (FLU), gentamicin (GNT), uric acid (UA), sulfamethoxazole (SFT), and ivermectin (IVT). Although FLU and IVT are veterinary pharmaceuticals, their presence in surface waters has been associated with agricultural and livestock activities,^52^ which justifies their inclusion in the interference study. The experiments were performed using solutions containing 5 μmol L^–1^ TA in the presence of each interferent at concentration ratios of 1:1 and 1:5 (analyte:interferent), as shown in Figure S9. These ratios were selected using a conservative approach, allowing the influence of interferents present at concentrations equal to or five times higher than that of TA to be assessed. A signal variation of ±10% was adopted as the criterion for significant interference. Under these conditions, GNT, UA, and SFT produced signal variations below 10%, indicating negligible interference. This behavior may be attributed to differences in their chemical structures and electrochemical properties compared with TA, which can limit their interaction with the electrode surface and their contribution to the analytical signal under the experimental conditions employed. Therefore, even at concentrations equal to or five times higher than that of TA, these species did not significantly affect the sensor response. A different behavior was observed for IVT and FLU, which produced decreases in the analytical signal, resulting in relative responses of 78% and 85%, respectively, at the highest tested ratio (see Figure S9B–D). These decreases correspond to signal reductions of approximately 22% and 15%, respectively, and therefore exceed the predefined ±10% criterion for significant interference.

This effect may be associated with partial overlap of the oxidation potentials and possible competitive interactions at the electrode surface, including adsorption processes, which may contribute to the observed decrease in signal. These results indicate that IVT and FLU can interfere with the determination of TA when present at sufficiently high relative concentrations and should therefore be considered when applying the sensor to samples containing these compounds.

Surface modification strategies could potentially minimize this interference by introducing more selective interaction sites or adjusting the surface chemistry to favor the interaction of TA over other compounds present in the sample. Overall, the sensor showed negligible interference from GNT, UA, and SFT under the investigated conditions, whereas a measurable interference was observed for IVT and FLU at the highest tested ratio. Thus, the selectivity toward TA should be considered satisfactory for the interferents that remained within the ±10% criterion, while the potential contribution of IVT and FLU should be taken into account in environmental samples.

Importantly, recovery tests were performed to evaluate the accuracy and applicability of the proposed method to real matrices, since the TA levels in the collected water samples were likely below the LOD. For this purpose, river water (see Figure S10) and tap water samples (Figure S11) were spiked at two concentration levels (2.0 and 3.5 μmol L^–1^) and analyzed using the standard addition calibration method. The results obtained are summarized in Table 2, showing recovery values in the range of 92–105%. These results indicate that the proposed sensor provides satisfactory accuracy and reliability within the investigated concentration range.

**Table 2:** TA Determination in River and Tap Water Using the CB/PLA-PT (n = 3)

Samples	Spiked/μmol L^–1^	Found/μmol L^–1^	Recovery/%
River water	2.00	1.97 ± 0.05	98 ± 2.5
	3.50	3.68 ± 0.04	105 ± 1.1
Tap water	2.00	1.95 ± 0.02	97 ± 1.0
	3.50	3.23 ± 0.12	92 ± 3.4

Table 3 compares the analytical performance and practical characteristics of the proposed CB/PLA-PT sensor with previously reported approaches for the determination of TA. As noticed, the GCE/PABA sensor presents a lower LOD (0.14 μmol L^–1^) and a linear range from 0.6 to 6.0 μmol L^–1^ also using DPV. However, these additional preparation steps may also limit the ease of fabrication and scalability of the sensor, particularly when a larger number of electrodes is required. Such modifications usually involve drop-casting procedures that require the preparation of dispersions, often using organic solvents, followed by drying steps that can be time-consuming and may result in less reproducible coatings on the electrode surface.

**Table 3:** Analytical Methods Reported in the Literature for TA Determination Compared to the Proposed Strategy Using CB/PLA-PT Platform^a^

Platform	Technique	Linear range/μmol L^–1^	LOD/μmol L^–1^	Sample	Reference
GCE/PABA	DPV	0.6–6.0	0.14	Tablets and urine	^1^
CB/PLA-PT	DPV	1.91–20.1	0.54	River and tap water	This work
	MI/UV–Vis	0.038–0.268	0.007	Pharmaceutical and veterinary formulations	^53^
	UA-DLLME/UHPLC-MS/MS	0.004–0.76	0.00029	Drinking, running, river, influent and effluent water	^54^
	Fe_3_O_4_/MIL-101(Cr)/UHPLC-MS/MS	0.00038–0.096	0.00011	Tap, river, pharmaceutical, hospital, influent, and effluent waters	^55^

a **DPV:** differential pulse voltammetry**; Fe**_**3**_ **O**_**4**_: magnetite nanoparticles supported on the chromium-based MOF MIL-101­(Cr**); GCE:** glassy carbon electrode**; HPLC:** high-performance liquid chromatography**; MI:** molecular iodine**; PABA:** modified para-aminobenzoic acid**; RP:** reverse phase**; UA-DLLME:** ultrasound-assisted dispersive liquid–liquid microextraction; **UHPLC-MS/MS:** ultrahigh performance liquid chromatography with tandem mass spectrometry**; UV–Vis:** ultraviolet–visible.

In contrast, the developed sensor combines additive manufacturing with rapid, reagent-free plasma activation, requiring only 120 s for surface treatment. This combination simplifies electrode fabrication and avoids the preparation and deposition of additional modifying materials. Although the LOD obtained (0.54 μmol L^–1^) is slightly higher, the proposed sensor exhibits a wider linear range (1.91–20.1 μmol L^–1^), which is suitable for environmental monitoring applications. More importantly from a practical perspective, the proposed approach requires only a simple dilution step prior to analysis, without the extraction or preconcentration procedures required by several chromatographic methods. In addition, the sensor showed good applicability in more complex matrices, such as river and tap water samples, requiring only a simple dilution step prior to analysis. This minimal sample pretreatment represents an important practical advantage for routine and decentralized measurements. As also shown in Table 3, chromatographic methods coupled with extraction and preconcentration strategies achieve substantially lower LODs than the electrochemical approaches. For instance, the combination of UA-DLLME with UHPLC-MS/MS enabled the determination of TA at subnanomolar levels, with a LOD of 2.9 × 10^–4^ μmol L^–1^. This remarkable detectability is mainly attributed to the preconcentration effect provided by the ultrasound-assisted dispersive liquid–liquid microextraction step, followed by the high selectivity and sensitivity of tandem mass spectrometric detection. Similarly, the Fe_3_O_4_/MIL-101­(Cr)/UHPLC-MS/MS method achieved even lower concentration levels through the use of magnetic solid-phase extraction based on a MOF-derived sorbent, which effectively concentrates the analyte before chromatographic separation and detection.

Despite their excellent analytical performance, these methodologies involve greater operational complexity, requiring multiple sample preparation steps, specialized extraction materials, and sophisticated instrumentation. In the case of UA-DLLME/UHPLC-MS/MS, the extraction procedure involves the use of organic solvents, ultrasonic dispersion, phase separation, and analyte enrichment prior to chromatographic analysis. Likewise, the Fe_3_O_4_/MIL-101­(Cr)/UHPLC-MS/MS approach relies on the synthesis or acquisition of a magnetic MOF sorbent, magnetic separation procedures, analyte desorption, and subsequent UHPLC-MS/MS analysis. Such requirements increase the overall analysis time, operational costs, and dependence on laboratory infrastructure. In contrast, the proposed electrochemical sensor allows the direct determination of TA in water samples with minimal sample pretreatment, requiring only dilution before measurement. Therefore, although chromatographic methods offer superior sensitivity, the developed CB/PLA-PT platform represents an attractive alternative for rapid and decentralized monitoring applications.

Nevertheless, some limitations of the proposed platform should also be considered. Although PLA-based 3D-printed electrodes provide advantages in terms of accessibility, scalability, and geometric flexibility, prolonged exposure to aqueous environments may lead to moisture uptake by the polymer matrix, which could potentially affect the stability and interfacial properties of the electrodes over extended storage or use.^56^ Appropriate drying or conditioning procedures may therefore be required to ensure consistent electrode performance. In addition, although plasma treatment provides a rapid and reagent-free approach for surface activation, the use of a hand-held plasma pen introduces an operator-dependent step, as variations in the distance, angle, and movement of the plasma source may influence the resulting surface properties. Consequently, the reproducibility of independently fabricated and treated electrodes could be further improved through the use of an automated plasma treatment system with controlled operating parameters.^38^ Long-term storage stability, interelectrode reproducibility, and performance in more complex environmental matrices should also be systematically investigated in future studies.^57^ Despite these limitations, the combination of 3D printing and rapid plasma activation remains a promising strategy for the development of low-cost, scalable, and potentially decentralized electrochemical sensors.

## 4 Conclusion

In this study, a CB/PLA-PT electrode was developed and evaluated for the electrochemical oxidation and quantification of TA. The atmospheric plasma treatment significantly improved the interfacial properties of the printed composite, leading to lower charge-transfer resistance, reduced peak-to-peak separation, and enhanced current response. These results indicate that plasma activation effectively increases the accessibility of electroactive CB sites and facilitates electron transfer at the electrode surface. In addition, the proposed 3D-printed electrode exhibited good linearity in the concentration range of 1.91–20.1 μmol L^–1^, with a sensitivity of 0.134 μA L μmol^–1^ and a LOD of 0.54 μmol L^–1^. Interestingly, the voltametric method demonstrated satisfactory repeatability and reproducibility, and its applicability was confirmed through the analysis of spiked environmental water samples. Overall, the results demonstrate that plasma activation is a reagentless, portable, and effective tool for improving the electrochemical activity of 3D-printed carbon-based electrodes, offering promising opportunities for the development of enhanced next-generation electrochemical sensors aimed at detection of emerging contaminants.

## Supplementary Material



## Notes

Further experimental details, including the delimitation of the electrode geometric area; morphological and wettability characterization; optimization of the plasma-treatment and voltammetric conditions; electrochemical characterization; repeatability and reproducibility assessments; interference studies; and determination of TA in river and tap water samples (PDF)
