# Supplementary material for: Plasma-Activated 3D-Printed Electrode for the Determination of Tolfenamic Acid in Environmental Samples
Source: ACS Omega. 2026 Oct 29;11(44):69013–25. doi: 10.1021/acsomega.6c08647 (PMC13756692; doi:10.1021/acsomega.6c08647)
Supplement: Supplementary file 1 [file ao6c08647_si_001.pdf]

Supporting Information

Plasma-activated 3D-printed electrode for the determination of tolfenamic acid in environmental samples

Cassiano Cunha de Souza[1*], Francisco Walison Lima Silva[2], Anne Alves Macedo [2], Dayenny Louise D`Amato[4] Célia Machado Ronconi[4], Wallace Burger Veríssimo[1], Raquel G. Rocha[5], Lucas V. de Faria[2], Fernando Henrique Cincotto[2,3], Rodrigo Alejandro Abarza Muñoz[6], Maria Auxiliadora Costa Matos[1] Renato Camargo Matos[1*]

[1] Department of Chemistry, Federal University of Juiz de Fora, Juiz de Fora, MG 36036-900, Brazil

[2] Institute of Chemistry, Federal University of Rio de Janeiro, Rio de Janeiro, RJ 21941-909, Brazil

[3] National Institute of Science and Technology of Bioanalytics – Lauro Kubota (INCTBio-LK), Institute of Chemistry, University of Campinas, Campinas, SP 13083-970, Brazil

[4] Institute of Chemistry, Fluminense Federal University, Niterói, RJ 24020-141, Brazil

[5] Department of Fundamental Chemistry, Institute of Chemistry, University of São Paulo, São Paulo, SP 05508-000, Brazil

[6] Institute of Chemistry, Federal University of Uberlândia, Uberlândia, MG 38408-100, Brazil

*Corresponding authors: renato.matos@ufjf.br and cassianosouza1990@gmail.com

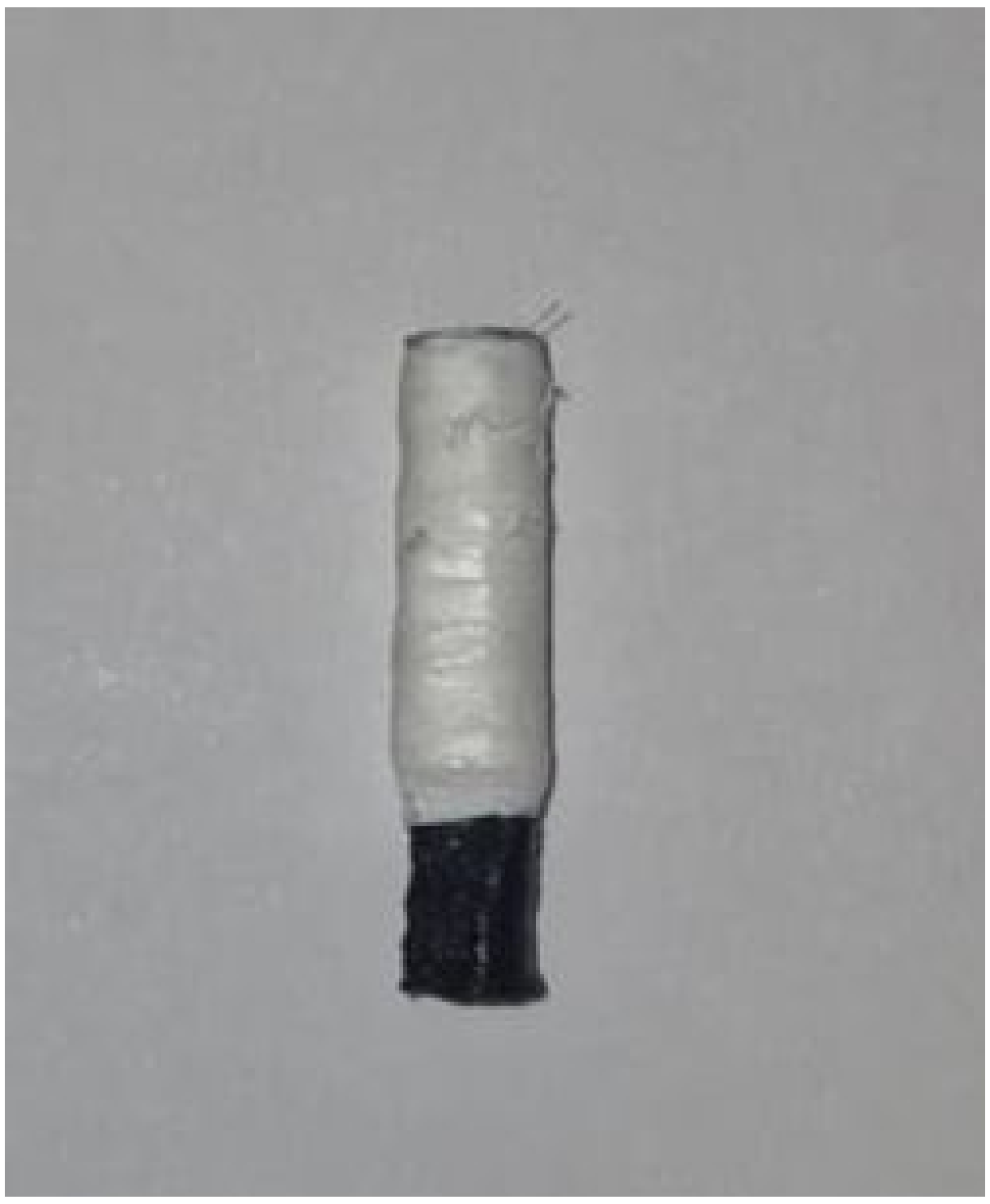

Figure S1: CB/PLA printed electrode after insulation with Teflon tape.

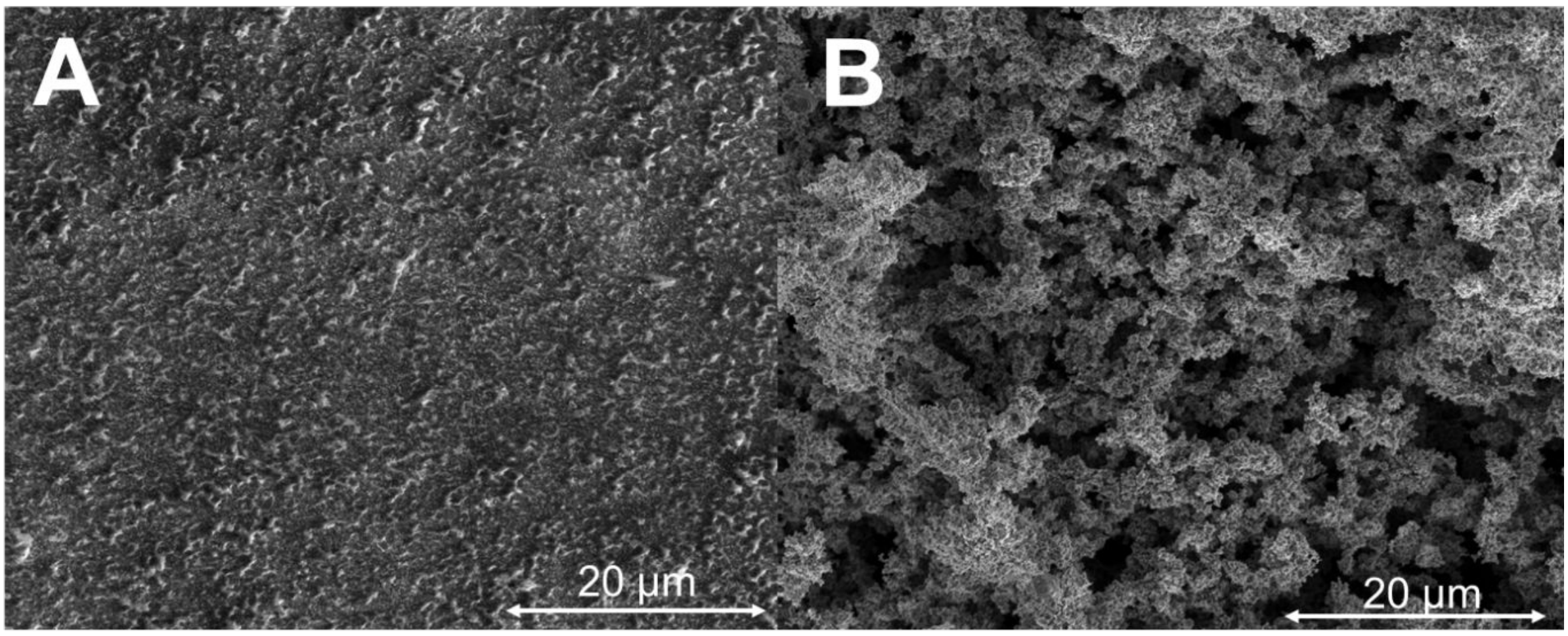

Figure S2: SEM images of the (A) CB/PLA and (B) CB/PLA-PT electrodes with HFW 64 µm

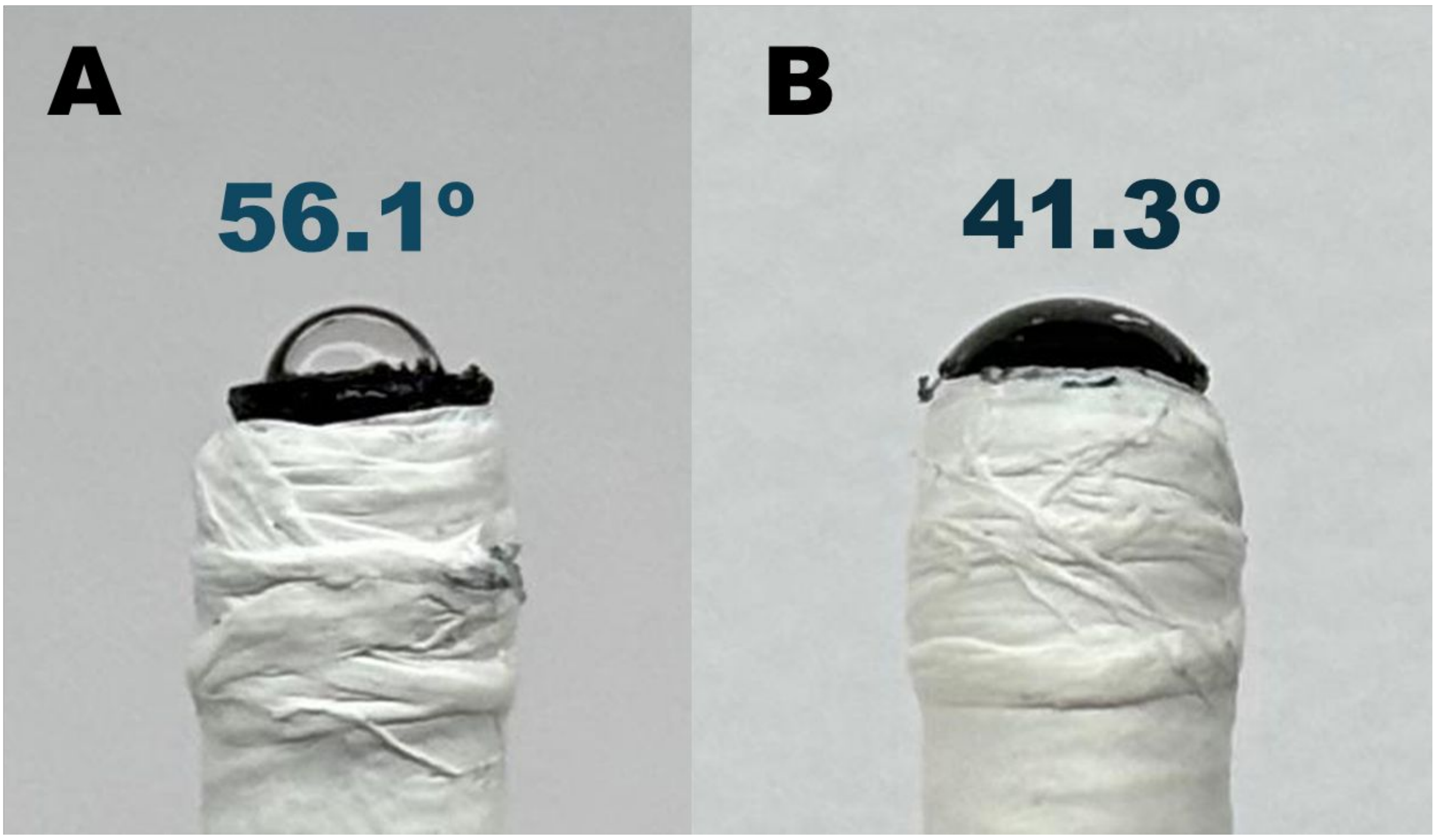

Figure S3: Water contact angles for the (A) CB/PLA and (B) CB/PLA-PT electrodes.

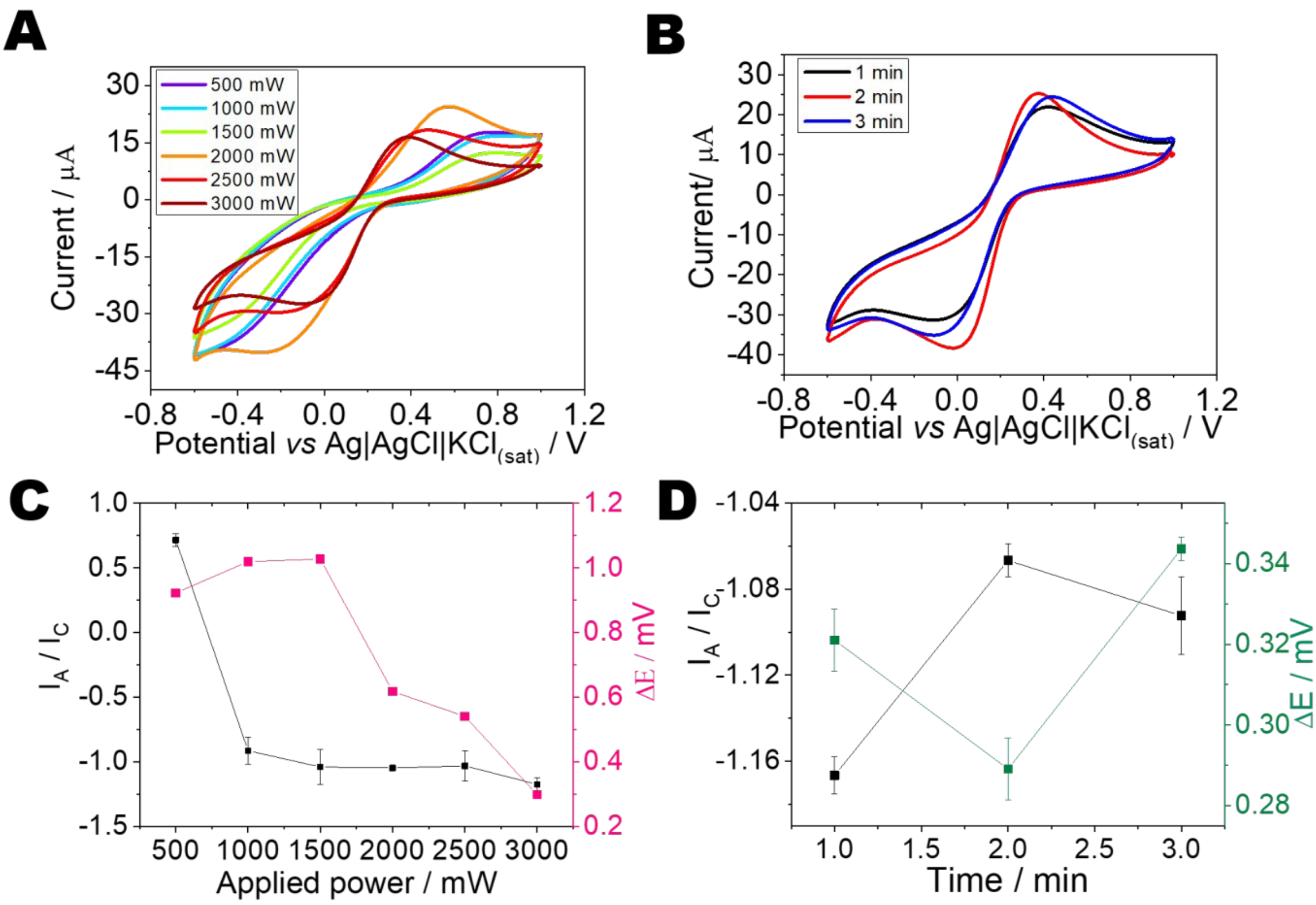

Figure S4: Cyclic voltammograms recorded in a solution containing 1 mmol L^{-1} $K_3[Fe(CN)_6]$ in 100 mmol L^{-1} KCl using the 3D-CB/PLA electrode after plasma treatment under different conditions. (A) Influence of applied plasma power (500–3000 mW). (B) Influence of plasma application time (1–3 min). CV conditions: scan rate: 50 mV; step potential: 5 mV. (C) Graph of the I_A / I_C and ΔE vs applied power. (D) Graph of the I_A / I_C and ΔE vs time

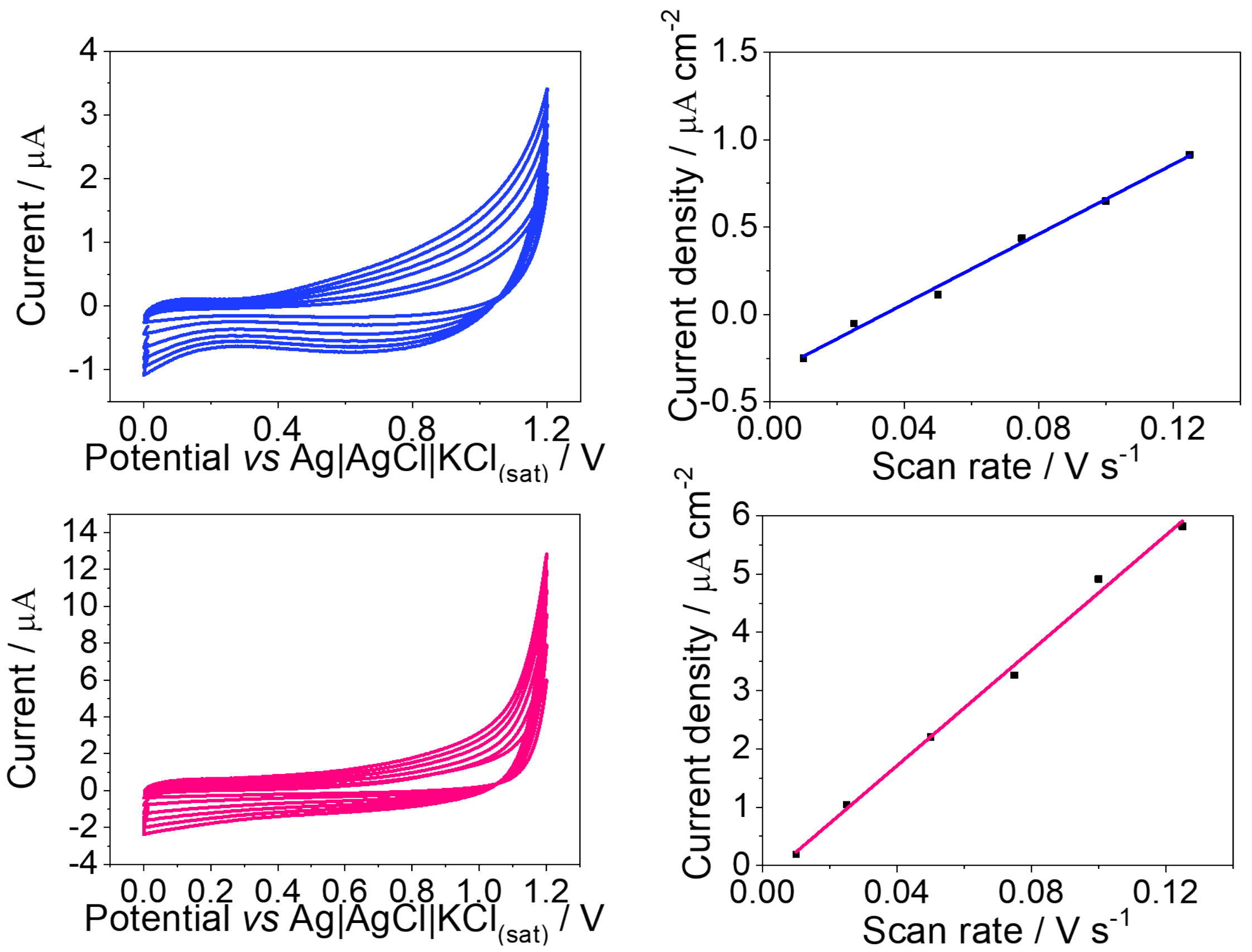

Figure S5: (A) Cyclic voltammograms recorded in 3.0 mol L^{-1} KCl solution within the potential window from 0.0 to 1.2 V for the CB/PLA electrode at different scan rates (10–125 mV s^{-1}). (B) Plot of current density versus scan rate for the CB/PLA electrode. (C) Cyclic voltammograms recorded in 3.0 mol L^{-1} KCl solution within the potential window from 0.0 to 1.2 V for the CB/PLA-PT electrode at different scan rates (10–125 mV s^{-1}). (D) Plot of current density versus scan rate for the CB/PLA-PT electrode.

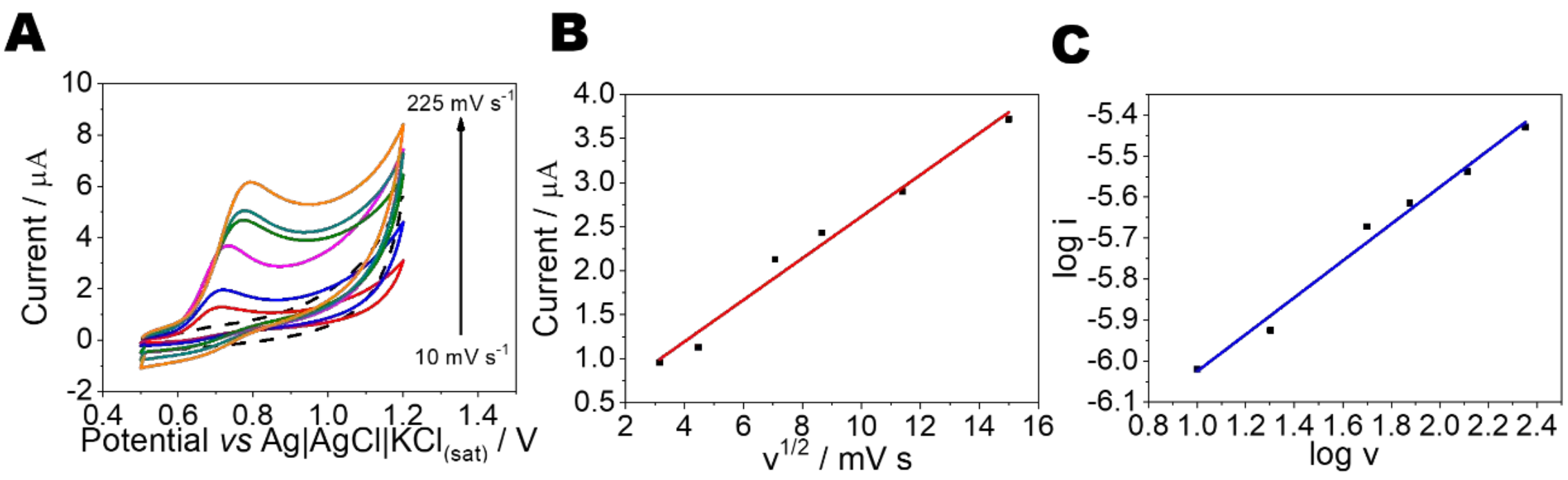

Figure S6: (A) CV of the TA (100 µmol L^{-1}) solution in BR buffer (pH 7.0) with scan rates ranging from 10 to 225 mV s^{-1} on the CB/PLA-PT electrode. (B) Plot showcasing the linear relationship between Ip and v1/2. (C) Plot of the log I vs log v linear relationship.

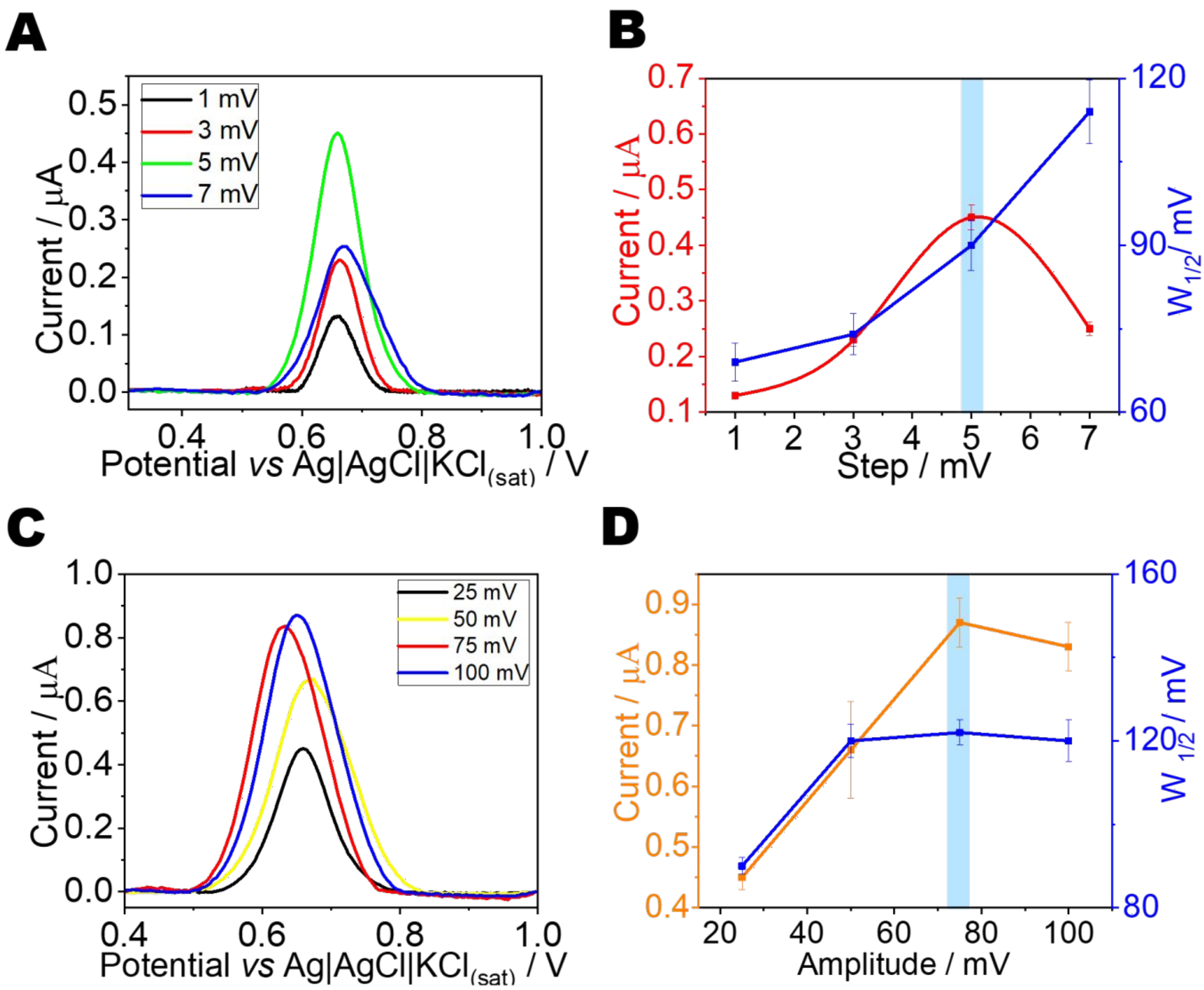

Figure S7: Optimization of differential pulse voltammetry (DPV) parameters for the determination of TA. (A) DPV responses obtained at different step potentials (1–9 mV). (B) Corresponding plot of peak current and half-wave potential as a function of the step potential. (C) DPV responses obtained at different pulse

amplitudes (25–100 mV). (D) Corresponding plot of peak current and half-wave potential as a function of the pulse amplitude. Measurements were carried out in BR buffer (pH 7.0).

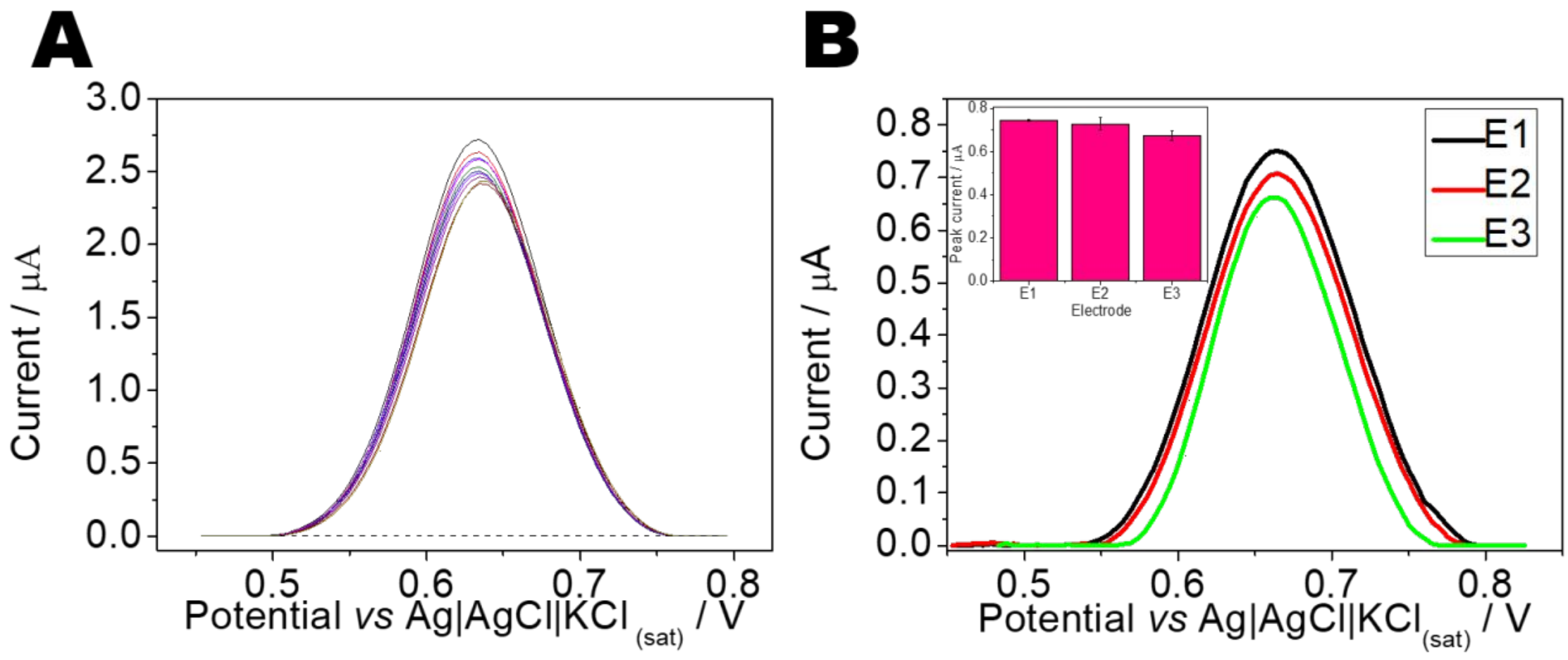

Figure S8: (A) DPV of TA (20.1 μmol L^{-1}) in BR buffer (pH 7.0) for the repeatability study (n = 9). (B) DPV of TA (5 μmol L^{-1}) measured by 3 different electrodes. Inset plot showcasing the peak current variation per electrode. DPV instrumental parameters: step potential: 5mV; amplitude: 75 mV; modulation time: 50 ms.

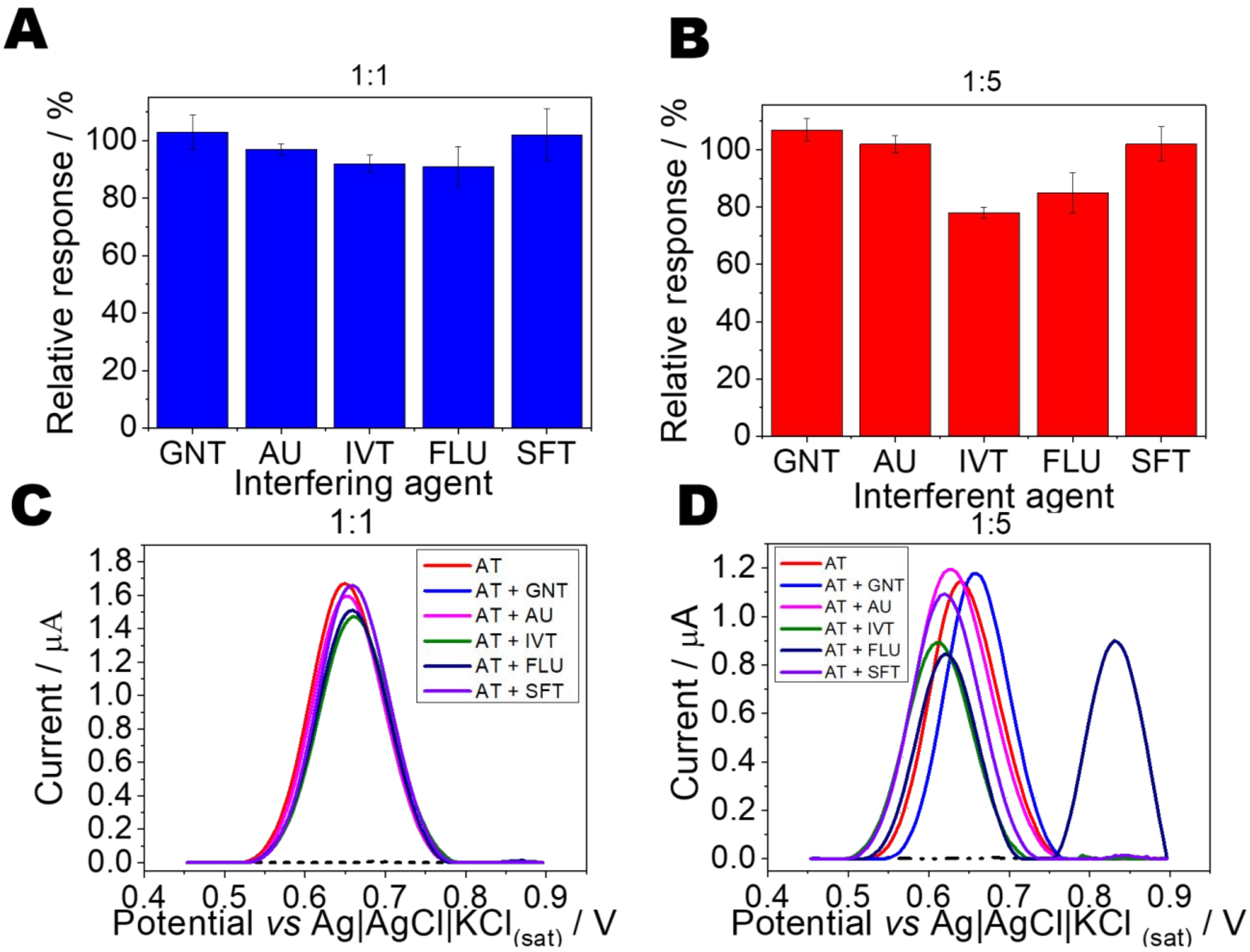

Figure S9: (A) Interference study with a 1:1 concentration ratio of TA: interferent agent. (B) Interference study with a 1:5 concentration ratio of TA: interferent agent. (C) DPV measurements from the 1:1 ratio experiment. (D) DPV measurements from the 1:5 experiment. Abbreviations: GNT: gentamicin; UA: uric acid; IVT: ivermectin; FLU: flunixin; SFT: sulfamethoxazole.

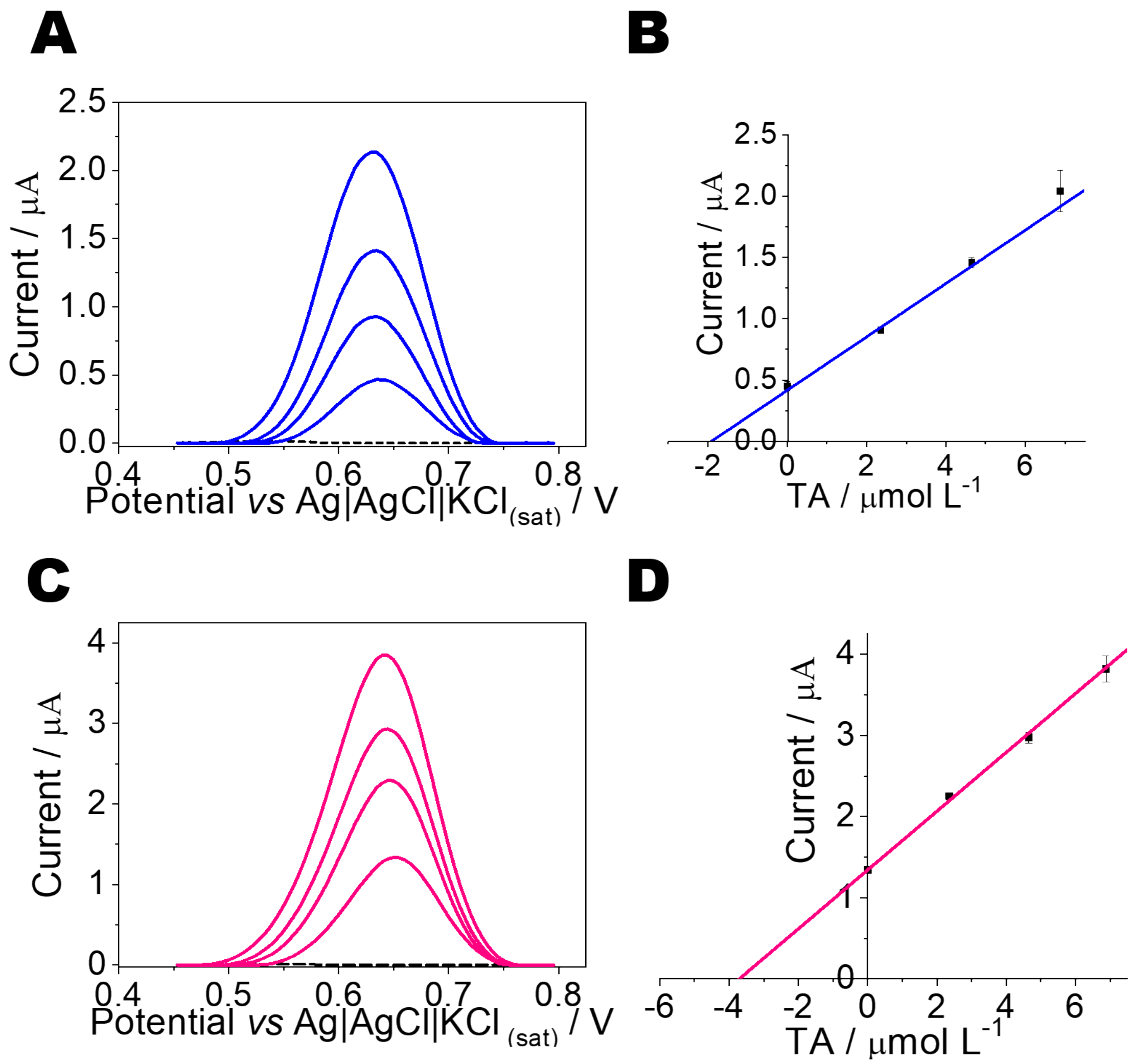

Figure S10: DPV recordings and corresponding standard addition calibration plots obtained for river water samples spiked with 2.0 µmol L^{-1} (A, B) and 3.5 µmol L-1 (C, D) of TA, followed by three successive additions of TA (2.4, 4.6 and 6.9 µmol L^{-1}), upon the CB/PLA-PT electrode. DPV conditions: step potential: 5 mV; amplitude: 75 mV; modulation time: 50 ms.

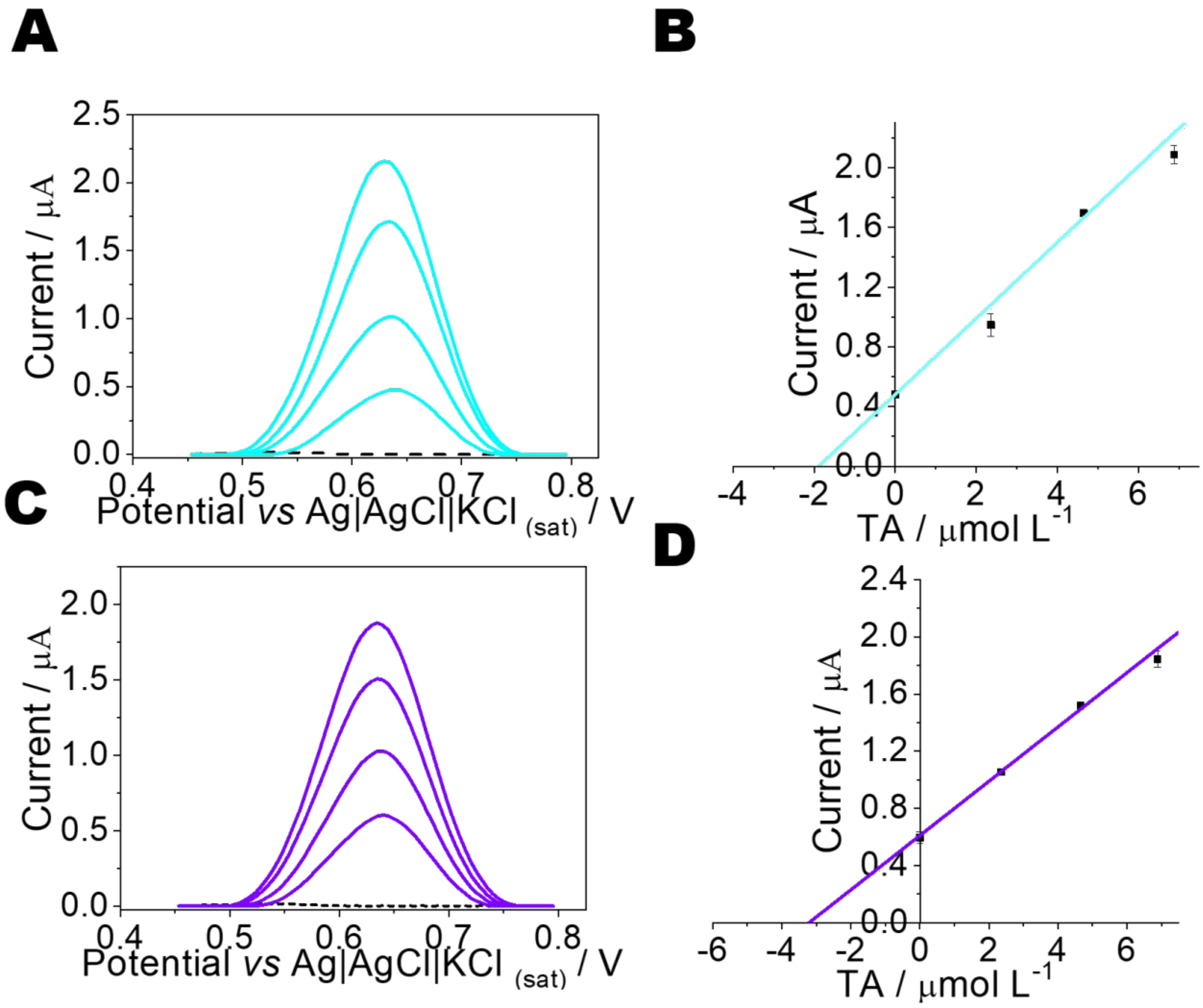

Figure S11: DPV recordings and corresponding standard addition calibration plots obtained for tap water samples spiked with 2.0 μmol L^{-1} (A, B) and 3.5 μmol L^{-1} (C, D) of TA, followed by three successive additions of TA (2.4, 4.6, and 6.9 μmol L^{-1}), using the CB/PLA-PT electrode. DPV conditions: step potential: 5 mV; amplitude: 75 mV; modulation time: 50 ms.
